# Antioxidant and Anti-Inflammatory Capacities of Fractions and Constituents from *Vicia tetrasperma*

**DOI:** 10.3390/antiox12051044

**Published:** 2023-05-04

**Authors:** Duc Dat Le, Kyung Hyun Min, Mina Lee

**Affiliations:** 1 College of Pharmacy, Research Institute of Life and Pharmaceutical Sciences, Sunchon National University, 255 Jungangno, Suncheon 57922, Republic of Korea; ddle@scnu.ac.kr; 2 School of Pharmacy and Institute of New Drug Development, Jeonbuk National University, Jeonju 54896, Republic of Korea; khmin1492@jbnu.ac.kr

**Keywords:** *Vicia tetrasperma*, antioxidant, anti-inflammatory, molecular docking, analytical method

## Abstract

The young leaves and shoots of *V*. *tetrasperma* are consumed daily as cooked vegetables and can provide various health benefits. The antioxidant and anti-inflammatory capacities of its total extract and fractions were accessed for the first time in this study. The bioactivities guided the separation of the active fraction (EtOAc), leading to the identification of nine flavonoid glycoside compositions from this plant for the first time. In addition, the fractions and all isolates were evaluated for their inhibition against NO and IL-8 production in LPS-stimulated RAW264.7 and HT-29 cell lines, respectively. The most active ingredient was further assayed for its inhibitory abilities to iNOS and COX-2 proteins. Indeed, its mechanisms of action modes were confirmed by Western blotting assays through the reduction in their expression levels. An in silico approach revealed the substantial binding energies of docked compounds into established complexes to verify their anti-inflammatory properties. In addition, the presence of active components in the plant was validated by an established method on the UPLC-DAD system. Our research has boosted the value of this vegetable’s daily use and provided a therapeutic approach for the development of functional food products for health improvement regarding the treatment of oxidation and inflammation.

## 1. Introduction

Antioxidants and inflammatory inhibitors are served as therapeutic interventions for the treatment of disease development. For example, antioxidant therapy could delay the progression of atherosclerotic lesions [1]. Antioxidants are also able to regress pre-malignant lesions or inhibit them from developing into cancer by interfering with the metabolic activation of chemical carcinogens [2]. On the other hand, excessive levels of ROS cause protein and DNA damage, leading to inflammation or mutation [3]. In the same manner, inflammation releases many mediators and cytokine production. This progression directly results in the body’s cells and tissue injuries [4]. Therefore, anti-inflammatory inhibitors have been developed and widely used for inflammatory diseases, such as etanercept and toculizumab, for the treatment of rheumatoid arthritis by reducing TNF-α and IL-6 activities, respectively [5,6]. However, some current anti-inflammatory inhibitors may cause some adverse effects such as glucocorticoids [7]. Thus, it is necessary to find active and safe antioxidant and anti-inflammatory inhibitors for the treatment of diseases.

The Vicia genus belong to the Leguminosae family, which contains many plants that are sources of bioactive gradients and micronutrients that have potential health benefits. Among the species, *V*. *faba*, *V*. *sativa*, *V*. *sativum*, *V*. *parviflora*, and *V*. *tenuifolia* have shown antioxidant capacity [8,9,10,11], and *V*. *sativa*, and *V*. *faba* have shown a potent anti-inflammatory effect [11,12,13,14,15]. In particular, *Vicia tetrasperma* is widely distributed across grasslands, fields, cultivated areas, hill slopes, and roadsides. This plant provides valuable food, vegetable, and medicinal products that maintain human health and provide economic benefits for local residents and farmers. Given their high calorie content, they are particularly useful as fodder and forage resources for grazing animals or as a feed component for animals. Additionally, they are inexpensive and rich sources of protein and minerals when compared to other supplements [16]. Its young leaves and shoots are consumed as cooked vegetables [17]. In Korea, this plant is called the eolchigi-wandu vegetable, and it is usually slowly simmered and made into a delicious salad by mixing it with soy sauce, sesame oil, and garlic. In previous studies on this plant, the high concentrations of D-pinitol or D-chiro-inositol [18] inhibited the biosynthesis of the raffinose family oligosaccharides, which are considered anti-nutritional factors by flatulence in humans, and isolated isolectins also suppressed hemagglutination activity [19]. However, studies on the phytochemical and pharmacology of this plant are limited. Specifically, there has been no report on the chemical investigation and antioxidant and anti-inflammatory properties from this vegetable in Korea.

Our primary screening revealed that the total extract of this plant had considerable antioxidant and anti-inflammatory effects. To the best of our knowledge, there is no report on the antioxidant and anti-inflammatory properties of the total extract and fractions previously derived from this plant. The aim of the current study is to access the bioactivities of the crude extract, fractions, and key isolated flavone glycosides; this will be achieved using methodologies to clarify a possible link between this herb and its observed anti-inflammatory and antioxidant potential. The active ingredients were obtained, evaluated for in vitro or Western blotting activities, and further proved by in silico studies. Then, an analytical method was established to validate the active components of this plant.

## 2. Materials and Methods

### 2.1. Plant Materials

The plant of *V*. *tetrasperma* (L.) Schreb. was collected from a herbal garden at Sunchon National University (Suncheon, Korea) in June, 2021, and was identified by Professor Mina Lee (College of Pharmacy, Sunchon National University). A voucher specimen (SCNUP 34) was deposited at the laboratory of Pharmacognosy, College of Pharmacy, Sunchon National University, Suncheon-si, Jeonnam-do, Korea.

### 2.2. Extraction and Separation of Compounds (**1**–**9**)

This plant was dried in the shade at room temperature. This dried material (1.5 kg) was ground into powder and was extracted with 100% ethanol (4 L × 5 times) in 1.5 h by sonification at room temperature. The solution was dried under vacuum to obtain a concentrated total extract. This total extract was then suspended in water (1 L), and successfully partitioned with *n*-hexane (1 L × 4 times, 14.50 g), CH_2_Cl_2_ (1 L × 4 times, 3.80 g), EtOAc (1 L × 4 times, 3.90 g), and *n*-BuOH (1 L × 4 times, 26.19 g). The active EtOAc fraction was performed to obtain nine isolates (**1**–**9**) by using multiple chromatographic techniques. The EtOAc fraction (4 g) was subjected to an open-column chromatography over ODS silica gel (400 g) column (5.5 cm × 24 cm) using multiple step gradients consisting of H_2_O: MeOH (from 8:2 to 0:10, each 1 L) to obtain eleven sub-fractions (1–11). Sub-fraction E3 (56 mg) was isolated on a preparative high-performance liquid chromatography (HPLC) over ODS column (250 × 20 mm, 5 μm) using a gradient solvent system consisting of H_2_O: CH_3_CN (from 8:2 to 7:3, *v*:*v*) to afford Compounds **1** (10 mg, *t*_R_ 20 min) and **2** (10 mg, *t*_R_ 40 min). Similarly, sub-fraction E5 (500 mg) was chromatographed on an HPLC over ODS column (250 × 10 mm, 5 μm) eluted with a gradient solvent system consisting of H_2_O: CH_3_CN (from 7:3 to 6:4, *v*:*v*) to afford Compounds **3** (4 mg, *t*_R_ 20 min), **4** (3 mg, *t*_R_ 25 min), **5** (2 mg, *t*_R_ 40 min), and **6** (2 mg, *t*_R_ 60 min), whereas Compounds **7** (3 mg, *t*_R_ 60 min), **8** (3 mg, *t*_R_ 80 min), and **9** (2 mg, *t*_R_ 105 min) were isolated from sub-fraction E4 (143 mg) by using an open column using Sephadex LH-20 (3.3 cm × 33 cm) with an isocratic solvent system consisting of H_2_O: MeOH (1:1, *v*:*v*), and further purified on an HPLC chromatographed over ODS column (250 × 10 mm, 5 μm) using isocratic solvent system consisting H_2_O: CH_3_CN (8:2, *v*:*v*).

### 2.3. UPLC-ESI-QTOF-MS Assay

The mass fragmentation of isolated compounds (**1**–**9**) was performed on a Waters UPLC system (Waters, Milford, MA, USA) coupled with Waters column and a Xevo G2-XS Q-TOF MS with electrospray ionization source (Waters, Milford, MA, USA). Triple TOF MS equipped with a DuosprayTM ion source was used to complete the high-resolution experiment. For mass detection, the instrument was operated in the negative ion electrospray mode, and the conditions of the MS/MS detector were as follows: the de-solvation gas was 800 L/h at 400 °C, the cone gas was 50 L/h, the source offset voltage was 80 eV, and the source temperature was 120 °C. A full scan was run in the negative mode with a mass range from *m*/*z* 50 to 1250 amu. The capillary voltage was 3.0 kV, and the sampling cone voltage was 40 eV. Sodium formate was used for mass spectrometer instrument calibration in the resolution mode. Leucine encephalin, which generated the reference ion *(m/z* 554.2615 [M-H]-), was used to ensure accuracy throughout the mass spectrometry analysis. All data acquisition and processes were performed for qualitative analysis by using MassLynx V4.1 and UNIFI V1.9 (Waters, Milford, MA, USA). The gradient elution of the mobile phase was conducted following the above established gradient elution.

### 2.4. Spectroscopic Data of Compounds **1**–**7**

Compound **1**: yellowish powder; ^1^H NMR (DMSO-*d*_6_, 400 MHz): *δ*_H_ 7.45 (1H, dd, *J* = 8.4, 2.1 Hz, H-6′), 7.41 (1H, d, *J* = 2.1 Hz, H-2′), 6.90 (1H, d, *J* = 8.4 Hz, H-5′), 6.76 (1H, brs, H-8), 6.75 (1H, s, H-3), 6.42 (1H, brs, H-6), 5.35 (1H, brs, H-1‴), 5.18 (1H, d, *J* = 7.2 Hz, H-1″), 3.91 (1H, d, *J* = 9.4 Hz, H_2_-4‴), 3.65 (1H, d, *J* = 9.4 Hz, H_2_-4‴), 3.71 (1H, d, *J* = 9.9 Hz, H-2‴), 3.54 (1H, m, H-2″), 3.46–3.64 (overlap, H-3″, 5″, H-6″), 3.30 (overlap, H2-5‴), 3.15 (1H, m, H-4″). ^13^C NMR (DMSO-*d*_6_, 100 MHz): *δ*_C_ 182.0 (C-4), 164.6 (C-2), 162.7 (C-7), 161.2 (C-5), 157.0 (C-9), 150.0 (C-4′), 145.9 (C-3′), 121.4 (C-1′), 119.3 (C-6′), 116.1 (C-5′), 113.6 (C-2′), 108.8 (C-1‴), 105.4 (C-10), 103.2 (C-3), 99.4 (C-6), 98.1 (C-1″), 94.7 (C-8), 79.4 (C-3‴), 77.1 (C-5″), 76.8 (C-2‴), 76.1 (C-2″), 75.7 (C-3″), 74.1 (C-4‴), 69.8 (C-4″), 64.3 (C-5‴), 60.6 (C-6″). Mass *m*/*z* 579.1371 [M − H]^−^ (C_26_H_28_O_15_, calc. 579.1355).

Compound **2**: yellowish powder; 1H NMR (DMSO-*d*_6_, 400 MHz): *δ*_H_ 7.45 (1H, dd, *J* = 8.3, 2.3 Hz, H-6′), 7.41 (1H, d, *J* = 2.3 Hz, H-2′), 6.90 (1H, d, *J* = 8.3 Hz, H-5′), 6.79 (1H, d, *J* = 2.1 Hz, H-8), 6.75 (1H, s, H-3), 6.44 (1H, d, *J* = 2.1 Hz, H-6), 5.07 (1H, d, *J* = 7.2, Hz, H-1″), 3.71 (1H, d, *J* = 11.0 Hz, H_2_-6″), 3.51 (overlap, H-3″, H_2_-6″), 3.27–3.29 (overlap, H-2″, 5″), 3.10 (1H, m, H-4″). ^13^C NMR (DMSO-*d*_6_, 100 MHz): *δ*_C_ 182.0 (C-4), 164.6 (C-2), 163.0 (C-7), 161.2 (C-5), 157.0 (C-9), 150.1 (C-4′), 145.9 (C-3′), 121.4 (C-1′), 119.2 (C-6′), 116.0 (C-5′), 113.6 (C-2′), 105.4 (C-10), 103.2 (C-3), 99.9 (C-6), 99.6 (C-1″), 94.8 (C-8), 77.2 (C-3″), 76.4 (C-5″), 73.2 (C-2″), 69.6 (C-4″), 60.7 (C-6″). HR-ESI-MS *m*/*z* 447.0921 [M − H]− (C_21_H_19_O_11_, calc. 447.0933).

Compound **3**: yellowish powder. ^1^H NMR (DMSO-*d*_6_, 400 MHz): *δ*_H_ 7.96 (1H, d, *J* = 8.7 Hz, H-2′,6′), 6.94 (1H, d, *J* = 8.7 Hz, H-3′, 5′), 6.87 (1H, s, H-3), 6.81 (1H, d, *J* = 2.1 Hz, H-8), 6.43 (1H, d, *J* = 2.1 Hz, H-6), 5.35 (1H, brs, H-1‴), 5.15 (1H, d, *J* = 7.3 Hz, H-1″), 3.91 (1H, d, *J* = 9.4 Hz, H_2_-4‴), 3.73 (1H, m, H-2‴), 3.71 (1H, m, H_2_-6″), 3.66 (1H, d, *J* = 9.4 Hz, H_2_-4‴), 3.45-3.52 (4H, m, H-2″, 3″, 5″, H_2_-6″), 3.30 (1H, m, H-5‴). ^13^C NMR (DMSO-*d*_6_, 100 MHz): *δ*_C_ 182.1 (C-4), 164.3 (C-2), 162.7 (C-5), 161.4 (C-7), 161.2 (C-4′), 157.0 (C-9), 128.7 (C-2′, 6′), 121.05 (C-1′), 116.1 (C-3′,5′), 108.8 (C-1‴), 105.4 (C-10), 103.0 (C-3), 99.4 (C-6), 98.1 (C-1″), 94.8 (C-8), 79.3 (C-3‴), 77.1 (C-3″), 76.8 (C-2″), 76.1 (C-2‴), 75.7 (C-5″), 74.0 (C-4‴), 69.8 (C-4″), 64.2 (C-5‴), 60.5 (C-6″). HR-ESI-MS *m*/*z* 563.1428 [M − H]^−^ (C_26_H_27_O_14_, calc. 563.1406).

Compound **4**: yellowish powder; ^1^H NMR (DMSO-*d*_6_, 400 MHz): *δ*_H_ 7.46 (1H, d, *J* = 2.4 Hz, H-2′), 7.44 (1H, dd, *J* = 8.2, 2.4 Hz, H-6′), 6.89 (1H, d, *J* = 8.2 Hz, H-5′), 6.74 (1H, s, H-3), 6.73 (1H, d, *J* = 2.0 Hz, H-8), 6.41 (1H, d, *J* = 2.0 Hz, H-6), 5.35 (1H, brs, H-1‴), 5.21 (1H, d, *J* = 7.1 Hz, H1″), 4.40 (1H, d, *J* = 11.9 Hz, H_2_-6″), 4.10 (1H, d, *J* = 11.9, 6.9 Hz, H_2_-6″), 3.93 (2H, overlap, H-3″, 4‴), 3.73–3.80 (2H, overlap, H-2″, 5″), 3.66 (1H, d, *J* = 9.4 Hz, H-4‴), 3.50–3.52 (1H, m, H-2‴), 3.30 (overlap, H-5‴), 3.20 (overlap, H-4″). ^13^C NMR (DMSO-*d*_6_, 100 MHz): *δ*_C_: 181.9 (C-4), 167.9 (C-3⁗), 167.1 (C-1⁗), 164.6 (C-2), 162.4 (C-7), 161.2 (C-5), 157.0 (C-9), 150.0 (C-4′), 146.0 (C-3′), 121.4 (C-1′), 119.1 (C-6′), 116.0 (C-5′), 113.6 (C-2′), 108.8 (C-1‴), 105.5 (C-10), 103.2 (C-3), 99.4 (C-6), 97.9 (C-1″), 94.6 (C-8), 79.3 (C-3‴), 76.4 (C-3″), 76.1 (C-2″), 75.5 (C-2‴), 74.0 (C-4‴), 73.7 (C-5″), 69.8 (C-4″), 64.2 (C-5‴), 64.2 (C-6″), 41.2 (C-2⁗). HR-ESI-MS *m*/*z* 665.1373 [M − H]^−^ (C_29_H_29_O_18_, calc. 665.1359).

Compound **5**: yellowish powder; 1H NMR (DMSO-*d*_6_, 400 MHz): *δ*_H_ 7.57 (1H, dd, *J* = 8.5, 2.3 Hz, H-6′), 7.46 (1H, d, *J* = 2.3 Hz, H-2′), 7.10 (1H, d, *J* = 8.5 Hz, H-5′), 6.84 (1H, d, *J* = 2.0 Hz, H-8), 6.79 (1H, s, H-3), 6.43 (1H, d, *J* = 2.0 Hz, H-6), 5.35 (1H, brs, H-1‴), 5.18 (1H, d, *J* = 7.1 Hz, H-1″), 3.91 (1H, d, *J* = 9.4 Hz, H_2_-4‴), 3.95 (1H, m, H-2‴), 3.74 (1H, m, H-5″), 3.71 1H, m, H_2_-6″), 3.66 (1H, d, *J* = 9.4 Hz, H_2_-4‴), 3.45–3.50 (overlap, H-3″, 2‴, H_2_-6″), 3.30 (overlap, H-5‴), 3.17 (1H, m, H-4″). ^13^C NMR (DMSO-*d*_6_, 100 MHz): *δ*_C_ 182.0 (C-4), 164.2 (C-2), 162.8 (C-7), 161.2 (C-5), 157.0 (C-9), 151.4 (C-4′), 146.9 (C-3′), 122.9 (C-1′), 118.9 (C-6′), 113.1 (C-2′), 112.2 (C-5′), 108.9 (C-1‴), 105.5 (C-10), 103.9 (C-3), 99.6 (C-6), 98.1 (C-1″), 94.8 (C-8), 79.3 (C-3‴), 76.1 (C-5″), 77.0 (C-3″), 76.1 (C-2‴), 75.7 (C-2″), 74.0 (C-4‴), 69.8 (C-4″), 64.2 (C-5‴), 60.5 (C-6″), 55.8 (C-OCH3). HR-ESI-MS *m*/*z* 593.1509 [M − H]^−^ (C_27_H_29_O_15_, calc. 593.1512).

Compound **6**: yellowish powder; ^1^H NMR (DMSO-*d*_6_, 400 MHz): *δ*_H_ 7.96 (1H, d, *J* = 8.8 Hz, H-2′,6′), 6.94 (1H, d, *J* = 8.8 Hz, H-3′,5′), 6.87 (1H, s, H-3), 6.78 (1H, d, *J* = 2.0 Hz, H-8), 6.42 (1H, d, *J* = 2.0 Hz, H-6), 5.35 (1H, d, *J* = 2.0 Hz, H-1‴), 5.21 (1H, d, *J* = 7.2 Hz, H-1″), 4.39 (1H, d, *J* = 11.9, 2.0 Hz, H_2_-6″), 4.12 (1H, dd, *J* = 11.9, 6.9 Hz, H_2_-6″), 3.92 (1H, d, *J* = 9.4 Hz, H_2_-4‴), 3.78 (1H, m, H-5″), 3.75 (1H, m, H-2‴), 3.67 (1H, d, *J* = 9.4 Hz, H_2_-4‴), 3.48–3.57 (2H, m, H-3″, 2‴), 3.21 (1H, m, H-4″), 2.01 (3H, s, CH_3_). ^13^C NMR (DMSO-*d*_6_, 100 MHz): *δ*_C_ 182.0 (C-4), 167.9 (C-7″), 164.4 (C-2), 162.4 (C-4′), 161.4 (C-7), 161.2 (C-5), 157.0 (C-9), 128.7 (C-2′,6′), 121.0 (C-1′), 116.0 (C-3′,5′), 108.8 (C-1‴), 105.5 (C-10), 103.1 (C-3), 99.4 (C-6), 97.8 (C-1″), 94.7 (C-8), 79.3 (C-3‴), 76.5 (C-3″), 76.81 (C-2″), 76.1 (C-2‴), 75.5 (C-5″), 74.0 (C-4‴), 69.8 (C-4″), 64.2 (C-5‴), 64.0 (C-6″), 20.6 (C-OCH_3_). HR-ESI-MS *m*/*z* 605.1528 [M − H]^−^ (C_28_H_29_O_15_, calc. 605.1512).

Compound **7**: yellowish powder; ^1^H NMR (DMSO-*d*_6_, 400 MHz): *δ*_H_ 7.56 (1H, d, *J* = 2.1 Hz, H-2′), 7.55 (1H, dd, *J* = 8.5 Hz, 2.1, H-6′), 7.05 (1H, d, *J* = 8.0 Hz, H-5′), 6.84 (1H, s, H-3), 6.60 (1H, d, *J* = 2.0 Hz, H-8), 6.39 (1H, d, *J* = 2.0 Hz, H-6), 5.38 (1H, d, *J* = 1.7 Hz, H-1‴), 5.17 (1H, d, *J* = 7.5 Hz, H-1″), 4.45 (1H, d, *J* = 11.9, H_2_-6″), 4.04 (1H, dd, *J* = 7.3, 11.9, H_2_-6″), 3.93 (1H, d, *J* = 9.4, H_2_-4‴), 3.85 (3H, s, 6″-C-OCH_3_), 3.78 (1H, m, H-5″), 3.67 (1H, d, *J* = 9.4, H_2_-4‴), 3.75 (1H, dd, *J* = 1.5, 5.8, H-2‴), 3.50–3.56 (2H, m, H-2″, 3″), 3.30 (overlap, H-5‴), 3.19 (1H, m H-4″), 2.01. (3H, s, CH_3_). ^13^C NMR (DMSO-*d*_6_, 100 MHz): *δ*_C_ 182.0 (C-4), 167.9 (C-CH_3_CO), 164.5 (C-2), 162.6 (C-7), 161.1 (C-5), 157.1 (C-9), 151.4 (C-4′), 147.4 (C-3′), 122.9 (C-1′), 118.7 (C-6′), 113.2 (C-2′), 112.2 (C-5′), 108.8 (C-1‴), 105.6 (C-10), 103.9 (C-3), 99.7 (C-6), 98.1 (C-1″), 94.5 (C-8), 79.3 (C-3‴), 76.4 (C-3″), 76.1 (C-2‴), 75.6 (C-2″), 74.0 (C-5″), 73.9 (C-4‴), 69.9 (C-4″), 64.1 (C-5‴), 63.9 (C-6″), 55.7 (5′-OCH_3_), 20.3 (CH_3_). HR-ESI-MS *m*/*z* 635.1634 [M − H]^−^ (C_29_H_31_O_16_, calc. 635.1618).

Compound **8**: yellowish powder; ^1^H NMR (CD_3_OD, 400 MHz): *δ*_H_ 7.70 (2H, d, *J* = 8.9 Hz, H-2′, 6′), 7.27 (1H, d, *J* = 15.9, H-α), 6.88 (2H d, *J* = 8.9 Hz, H-3′, 5′), 6.84 (1H, d, *J* = 1.9 Hz, H-2⁗), 6.73 (1H, dd, *J* = 2.0, 8.2 Hz, H-6⁗), 6.61 (1H, d, *J* = 8.2 Hz, H-5⁗), 6.60 (1H, d, *J* = 2.0 Hz, H-8), 6.38 (1H, d, *J* = 2.0 Hz, H-6), 6.31 (1H, s, H-3), 6.01 (1H, d, *J* = 15.9 Hz, H-*β*), 5.50 (1H, brs, H-1‴), 5.18 (1H, d, *J* = 7.4 Hz, H-1″), 4.29 (1H, d, *J* = 9.7 Hz, H_2_-4‴), 4.22 (1H, d, *J* = 11.4 Hz, H_2_-5‴), 4.18 (1H, d, *J* = 11.4 Hz, H_2_-5‴), 3.92 (1H, m, H-2″), 3.86–3.88 (3H, m, H-2‴, H_2_-6″, 4‴), 3.66–3.76 (overlap, H-3″, H_2_-6″), 3.74 (3H, s, OCH_3_-3⁗), 3.74 (1H, m, H-2″), 3.68 (1H, m, H-3″), 3.56 (1H, m, H-5″), 3.43 (1H, m, H-4″). ^13^C NMR (CD_3_OD, 100 MHz): *δ*_C_ 183.8 (C-4), 168.7 (CO-5‴), 166.4 (C-2), 164.2 (C-7), 163.0 (C-4′), 162.8 (C-5), 158.8 (C-9), 150.6 (C-4⁗), 149.1 (C-3⁗), 146.9 (C-*β*), 129.5 (C-2′, 6′), 127.3 (C-6′), 123.0 (C-1⁗), 124.2 (C-6⁗), 116.9 (C-3′, 5′), 116.3 (C-5⁗), 114.3 (C-α), 111.1 (C-2⁗), 109.9 (C-1‴), 107.0 (C-10), 104.1 (C-3), 100.4 (C-1″), 99.6 (C-6), 95.9 (C-8), 79.2 (C-3‴), 78.7 (C-5″), 78.6 (C-2‴), 78.3 (C-3″), 77.3 (C-2″), 75.1 (C-4‴), 71.3 (C-4″), 67.8 (C-5‴), 62.4 (C-6″), 56.2 (3⁗-OCH_3_). HR-ESI-MS *m*/*z* 739.1878 [M − H]^−^ (C_36_H_35_O_17_, calc.739.1880).

Compound **9**: yellowish powder; ^1^H NMR (CD_3_OD, 400 MHz): *δ*_H_ 7.29 (1H, d, *J* = 15.9 Hz, H-*β*), 7.28 (1H, d, *J* = 2.0 Hz, H-2′), 7.25 (1H, dd, *J* = 8.2, 2.1 Hz, H-6′), 6.86 (1H d, *J* = 8.2 Hz, H-5′), 6.84 (1H, d, *J* = 1.8 Hz, H-2⁗), 6.75 (1H, dd, *J* = 8.0, 1.8 Hz, H-6⁗), 6.64 (1H, d, *J* = 8.1 Hz, H-5⁗), 6.60 (1H, d, *J* = 2.1 Hz, H-8), 6.40 (1H, d, *J* = 2.1 Hz, H-6), 6.29 (1H, s, H-3), 6.03 (1H, d, *J* = 15.9 Hz, H-α), 5.50 (1H, brs, H-1‴), 5.18 (1H, d, *J* = 7.4 Hz, H-1″), 3.75 (3H, s, 3⁗-OCH_3_), 3.47–3.91 (overlap, H-2″, 3″, 5″, 6″, 2‴, 4‴, 5‴), 3.40 (1H, m, H-4″). HR-ESI-MS *m*/*z* 755.1856 [M − H]^−^ (C_36_H_35_O_18_, calc. 755.1829).

### 2.5. Cell Culture and Cell Viability

Mouse macrophages (RAW264.7) and human colon epithelial (HT-29) cells were acquired from the Korean Cell Lines Bank (Seoul, Republic of Korea). These cell lines were grown at 37 °C and were maintained in Dulbecco’s modified Eagle’s medium (DMEM) supplemented with 10% heat-inactivated FBS, streptomycin sulfate (100 µg/mL), and penicillin (100 IU/mL) in a humidified atmosphere of 5% CO_2_. RAW264.7 cells were seeded into 96-well plates followed by preincubation for 24 h before addition with samples for 1 h, and then stimulation with LPS (1 μg/mL) for 16 h, whereas HT-29 cells were seeded into 96-well plates, preincubated for 24 h, then treated with 100 μM of compounds (**1**–**9**) for 2 h, and stimulated with LPS (100 ng/mL) for 12 h. The cultured cells were incubated with MTT (5 mg/mL) at 37 °C for four hours. The supernatants were removed and 100 µL dimethyl sulfoxide (DMSO) (SigmaAldrich, Saint Louis, MO, USA) was added. After five minutes, the absorbance of the formazan crystals was measured at 570 nm in a microplate reader (Bio Tek Instruments, Winooski, VT, USA).

### 2.6. Measurement of ROS Accumulation

RAW264.7 were seeded in a 6-well plate at concentration of 1 × 10^4^ cells/well for 20 h and then treated with positive control (ascorbic acid, A.A) and samples (total extract and fractions) at a concentration of 100 μg/mL. Subsequently, 10 μM dichlorodihydrofluorescein diacetate (DCFH-DA) diluted with 1/1000 in a serum-free medium was added after removing all the cell culture medium. After incubation for 20 min at 37 °C in dark, the cells were washed 3 times with PBS to remove DCFH-DA, which had not been entered into cells. The cells were visualized by an inverted fluorescence microscope and further detected by a fluorescent microplate reader at 485 and 530 nm.

### 2.7. Measurement of NO Production

The level of NO production was determined by measuring the amount of secreted nitrite from the cell culture supernatants, as described previously [20]. Briefly, RAW264.7 cells (1 × 10^5^ cells/well) were pretreated with samples (total extract, fractions, 100 μg/mL, and isolates **1**–**9**, 100 μM) for 1 h and stimulated with LPS (1 μg/mL) for 20 h. Then, the collected supernatant was incubated with an equal volume of Griess reagent by using equal volumes of 1% (*w*/*v*) sulfanilamide in 5% (*v*/*v*) phosphoric acid and 0.1% (*w*/*v*) N-(1-naphtyl) ethylenediamine at room temperature for 10 min. The absorption was measured at 550 nm by microplate reader (Bio-Tek Instruments, Inc., Winooski, VT, USA). The LPS-induced NO production was measured in the treated samples and controls (with and without LPS in the absence of sample addition).

### 2.8. DPPH and ABTS Assay

The scavenging abilities of samples on DPPH and ABTS radicals were assayed following a previous method [20] in which total extract and fractions were prepared at concentration of 100 μg/mL for the experiments. Trolox was considered a reference standard. The calibration curve was built using various concentrations of Trolox (0–100 μM). Results were presented as µmol of Trolox equivalent per gram of extract, and were compared to the Trolox standard curve.

### 2.9. Western Blotting

The reduction ability of iNOS and COX-2 protein expression on RAW264.7 cells of the strong Active Compound 6 and total extract was examined, following our previous study [21]. Briefly, RAW264.7 cells were seeded and cultured using the above-explained experimental conditions in 6-well plates for 24 h. Then, the cultured cells were treated with Compound **6** and total extract at concentrations of 100, 200 μM and 100, 200 μg/mL, respectively, for 1 h, then stimulated with LPS (1 μg/mL). After 24 h, (iNOS and COX-2) cells were washed with cold phosphate-buffered saline (PBS) twice. The cell lysates were extracted using a protein extraction solution (proprep, iNtRON, Biotechnology, Daejeon, Korea). The protein concentration was determined by the Bradford assay method. Equal amounts of control, LPS, and compound-treated samples (30 µg) were separated by 10% SDS-PAGE gels and transferred on PVDF membranes. The membranes were blocked with 5% skim milk powder in plain buffer [20 mM Tris-HCl (pH 7.4) and 4 M NaCl] for one hour at room temperature. The membranes were incubated with primary antibodies (iNOS, COX-2, and β-actin) at 4 °C overnight, and then washed three times with a wash buffer [1 M Tris-HCl (Ph 7.4), 4 M NaCl, Tween-20 in DW] for 10 min. They were incubated in specific secondary antibodies (1:2000 dilution) conjugated with horseradish peroxidase at RT for two hours and washed. The protein signals were obtained using chemiluminescence detection reagents (Thermo Fisher Scientifc, Waltham, MA, USA) and imaged using a bio-imaging system (MicroChemi 4.2 Chemilumineszenz-System, Modi’in-Maccabim-Re’ut, Israel).

### 2.10. Measurement of IL-8 Production

The IL-8 production assay was used on HT-29 cells (3 × 10^5^ cells/well) in 96-well plates using ELISA kit (BD OptEIATM, CA, USA) following the manufacturer’s instructions.

### 2.11. Molecular Docking

The 3D structures of iNOS (PDB ID: 3E7G), COX-2 (PDB ID: 5IKQ), and IL-8 (PDB ID: 5D14) proteins were obtained from the RCSB protein data bank (https://www.rcsb.org; accessed on 12 October 2022). Proteins and ligand were prepared using MGL tools 1.5.6. The structures of receptors were processed by removing water, adding polar hydrogen atoms, and Kollman charges. Structure (6″-acetylapiin, **6**) was downloaded from PubChem (https://pubchem.ncbi.nlm.nih.gov, 12 October 2022) in sdf formats, and structures (apigenin-7-*O*-[2″-*O*-(5‴-*O*-feruloyl)-D-apiofuranosyl]-D-glucopyranoside, **8**; luteolin-[2″-*O*-(5‴-*O*-feruloyl)-D-apiofuranosyl]-D-glucopyranoside, **9**) were prepared using by Avogadro package via force field method-MMFF94. Then, the geometries of these structures were transformed into pdbqt format using the Open Babel. The ligand conformations were performed by adding Gasteiger charges. A grid box of coordinates was determined using Pymol. A total of 100 runs were conducted under default parameters using the Lamarckian genetic algorithm. The protein–ligands docking calculations were performed using AutoDock 4.2. Residues–ligand interactions were visualized with Discovery studio 2021.

### 2.12. Method Validation

#### 2.12.1. UPLC-DAD System and Separation Condition

The sample was analyzed using a Waters UPLC system (Waters, Milford, MA, USA) equipped with an autosampler, column temperature controller, and DAD detector. A Waters Acquity UPLC HSS T3 column (2.1 × 100 mm, 1.8 µm) was used to carry out the experiment at 40 °C. The mobile phases were buffered with 0.1% formic acid for both aqueous (water, A) and organic (acetonitrile, B) elements. The flow rate was set to 0.4 mL/min, and the injection volume was 2 μL. The gradient elution of the mobile phase was conducted as follows: 0–1 min (B: 5 %), 1–4 min (B: 5–15%), 4–12 min (B: 15–35%), 12–17 min (B: 35–45%), 17–23 min (B: 45–100%). Then, it was held at 100% B for 3 min and then returned to the initial conditions for re-equilibration. The range of DAD detection wavelength was set to 200–400 nm and chromatograms were recorded at 254 nm.

#### 2.12.2. Detection of Wavelength

The marker compounds (**1** and **3**–**7**) were isolated from *V*. *teterasperma*, and further purified with purity of 93.3, 95.2, 98.0, 97.2, 95.3, and 97.4%, respectively.

Preparation of standard solutions: a standard mixture including analytes was dissolved in 100% methanol as a stock solution and then diluted with 100% methanol to obtain working concentrations for calibration curves. The solution was sealed by an elastic–plastic film and stored in the refrigerator at 4 °C for analysis.

#### 2.12.3. Preparation of Calibration Standard Solution

Sample preparation: a dried herb of *V*. *teterasperma* (1 g) was extracted with 100% ethanol (10 mL) by sonication for 90 min. The extracted solution was filtered through filter paper. Its solvent was removed at room temperature using an evaporator under nitrogen gas. For a quantitative analysis of the *V*. *terterasperma*, a total extract (13 mg) was dissolved in 1 mL of methanol and sonicated for 5 min. The sample solutions were then filtered through a 0.22 µm PTFE syringe filter and the filtrates were collected and stored at 4 °C prior to use.

Linearity, LODs, LOQs: the identification of standards in the sample was accessed by calculating relative retention time and UV absorption maxima in comparison to those of standard mixture at the same UPLC analytical conditions. The UPLC-DAD method was applied to conduct the experiment with validation parameters, including linearity, limit of detection (LOD), limit of quantification (LOQ), precision, and accuracy, in accordance with the guidelines described at the international conference on Harmonisation, whereas the calibration curves were built using the regression equation based on the peak areas (y) in response to the corresponding concentrations (x, µg/mL) of six markers in the standard solutions at eight different concentrations, and the correlation coefficients (*r*^2^) were calculated. The LOD values were calculated using LOD = 3.3 × SD/S. In addition, the LOQ values were calculated using LOQ = 10 × SD/S, while SD was the standard deviation and S was the slope of the calibration curve.

Intra- and inter-day precision were performed six times during the same day and on six successive days, respectively. The relative standard deviation (RSD) was obtained using the following equation: RSD (%) = SD × 100/mean measured concentration.

To prove the repeatability of the analytical method, the accuracy was experimented with by spiking the known amounts of the standards into a working solution. Their concentrations were prepared at three (high, medium, and low) levels such as Compounds **1** and **3** (250, 100, 62.5 ug/mL), Compounds **4** and **5** (60, 24, 15 ug/mL), and Compounds **6** (200, 50, 31.5 ug/mL) and **7** (20, 8, 5 ug/mL). Then, a recovery test was performed using the above analytical condition. The recovery (%) was calculated using the following equation:

Recovery (%) = [(Amount found − original amount) × 100]/spiked amount.

### 2.13. Statistical Analysis

Data were presented as mean ± SD (*n* = 3) of at least three replicates. The nonparametric one-way ANOVA followed by Turkey’s multiple comparison test using the Graphprism version 8.0.1 software (Graphpad Software, La Jolla, CA, USA) was used for statistical analyses. * *p* < 0.05, ** *p* < 0.01, compared to controls, were considered significant values.

## 3. Results

### 3.1. Antioxidant and Anti-Inflammatory Capacities of Total Extract and Fractions

**Figure 1:**
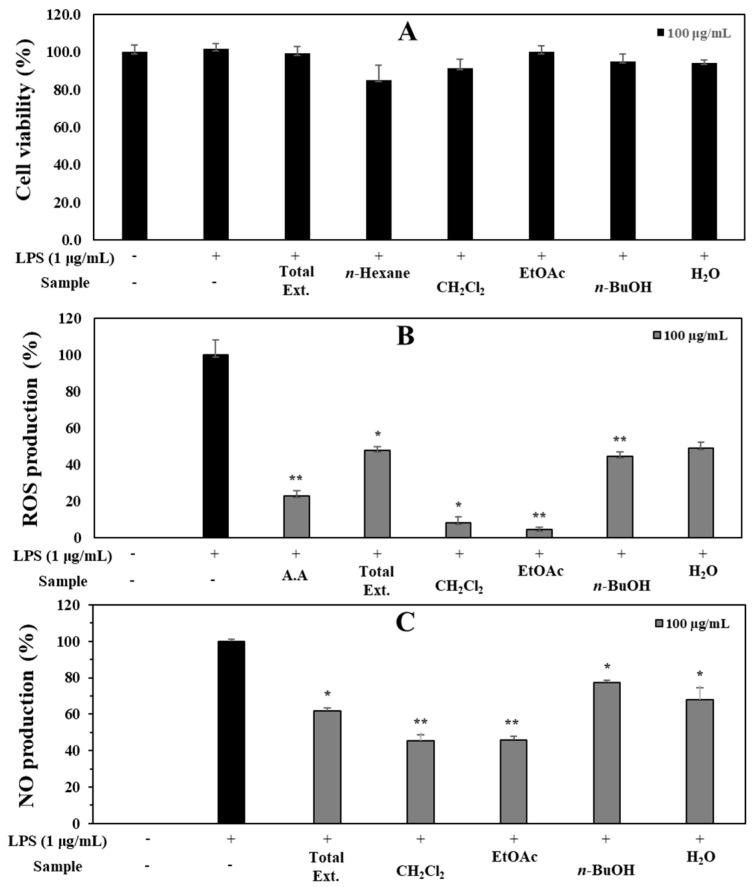
Cytotoxic (**A**), ROS production (**B**), and NO production (**C**), inhibitory effects of total extract (Total Ext.), and fractions of *V*. *tetrasperma* on RAW264.7 cells. Each experiment was performed in triplicate. The data are represented as mean ± SD. * *p* < 0.05, ** *p* < 0.01 vs. LPS-treated group.

The total extract of *V*. *tetrasperma* was screened for its antioxidant effect using an ROS assay [22] with some modifications by using RAW264.7 cells. The total extract of *V*. *tetrasperma* exhibited the significant inhibition of ROS production in LPS-mediated RAW264.7 cells with an inhibition rate of 52.1% at a concentration of 100 μg/mL, compared to the positive control (ascorbic acid, 76.9%), without cytotoxic effects (cell viability > 90%). Thus, the total extract was suspended in water and partitioned into *n*-hexane, CH_2_Cl_2_, EtOAc, *n*-BuOH, and distilled water (DW) fractions. The cell viability of the total extract and fractions were conducted on RAW264.7 cells. The result revealed that the *n*-hexane fraction showed some toxic effects on cells. Thus, this fraction was eliminated for the ROS experiment. Subsequently, CH_2_Cl_2_, EtOAc, *n*-BuOH, and DW fractions were examined for their antioxidant effect using an ROS assay. Among the tested samples, EtOAc and MC fractions showed potential for antioxidant effects with inhibition rates at 95.4% and 91.8%, respectively, stronger than those of positive control (ascorbic acid, 76.9%) at the tested concentration of 100 μg/mL. *n*-BuOH and DW fractions showed weak antioxidant effects (Figure 1). The abilities of the total extract and fractions were also evaluated for their anti-inflammatory effect against NO production. The EtOAc and CH_2_Cl_2_ fractions showed strong inhibition of NO production with suppressions of 45.5 and 45.9%, respectively, at 100 μg/mL. The *n*-BuOH and DW fractions displayed weaker inhibitory effects with inhibition rates of 30.5 and 22.5% (compared to those of the LPS-treated group), respectively, at 100 μg/mL. Additionally, the EtOAc showed the highest antioxidant capacities among all the fractions for both radical scavenging activities on DPPH^•^ and ABTS^•+^ (Table 1). Therefore, the strong active fraction (EtOAc) was selected as the material for separation.

### 3.2. Identification of Constituents

The most active fraction (EtOAc), which has potent antioxidant and anti-inflammatory capacities, was separated using multiple chromatographic methods to yield nine compounds (**1**–**9**) (Figure 2; Figures S1–S32).

**Figure 3:**
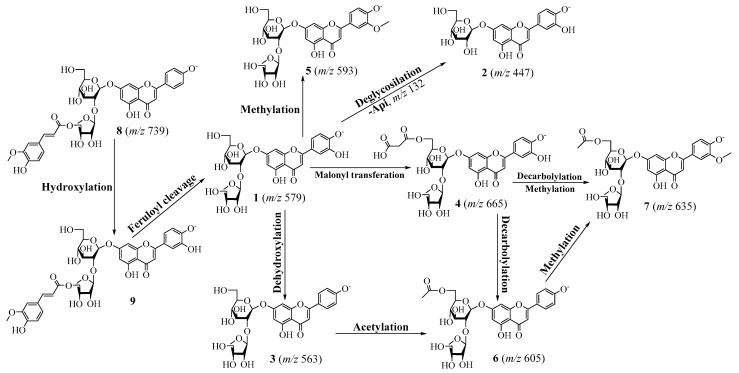
Proposed biogenetic pathway of flavonoid glycosides (**1**–**9**) from *V*. *teterasperma*.

Compound **1** was obtained as a yellowish powder. Its molecular formula was identified as C_26_H_28_O_15_ based on the HR-ESI-MS ion peak [M − H]^−^ at *m*/*z* 579.1371 (cal. for C_26_H_27_O_15_, 579.1355). ^1^H NMR spectrum of **1** displayed the signals of an ABX spin-coupling system at *δ*_H_ 7.45 (1H, dd, *J* = 8.4, 2.1 Hz, H-6′), 7.41 (1H, d, *J* = 2.1 Hz, H-2′), and 6.90 (1H, d, *J* = 8.4 Hz, H-5′), and an AX coupling system at *δ*_H_ 6.76 (1H, brs, H-8) and 6.42 (1H, brs, H-6), revealing a flavonol bearing an asymmetrical B-ring substitution. The HMBC cross-peak of H-3 (*δ*_H_ 6.75) to C-2 (*δ*_C_ 164.6)/C-4 (*δ*_C_ 182.0)/C-1′ (*δ*_C_ 121.4) together with an MS negative ion peak [M − H]^−^ at *m*/*z* 285 (Table 1) implied a luteolin aglycone of **1 [23]**. Subsequently, 15 carbon signals corresponding to the luteolin aglycone were successfully assigned using HSQC and HMBC spectra. In addition, two anomeric protons were observed at *δ*_H_ 5.35 (1H, brs, H-1‴) and 5.18 (1H, d, *J* = 7.2 Hz, H-1″), suggesting two glycoside units. Indeed, the MS peaks produced secondary dissociation mass fragment ion peaks [M − H − Api]^−^ at *m*/*z* 447 and [M − H − Api − Glc]^–^ at *m*/*z* 285 (Table 1; Figure 3), indicating a the reduction in apiofuranosyl and glucopyranosyl moieties. Moreover, the HMBC cross-peak of H-1″ (*δ*_H_ 5.18) to C-7 (*δ*_C_ 162.7), and those of H-1‴ (*δ*_H_ 5.35) to C-2″ (*δ*_C_ 76.1), confirmed the glucopyranosyl linked to luteolin aglycone through C-7 and apiopyranosyl attached to C-2″ of glucose unit. Thus, the structure of luteolin 7-*O*-[2-*β*-D-apiofuranosyl)-*β*-D-glucopyranoside of **1** was established.

**Figure 4:**
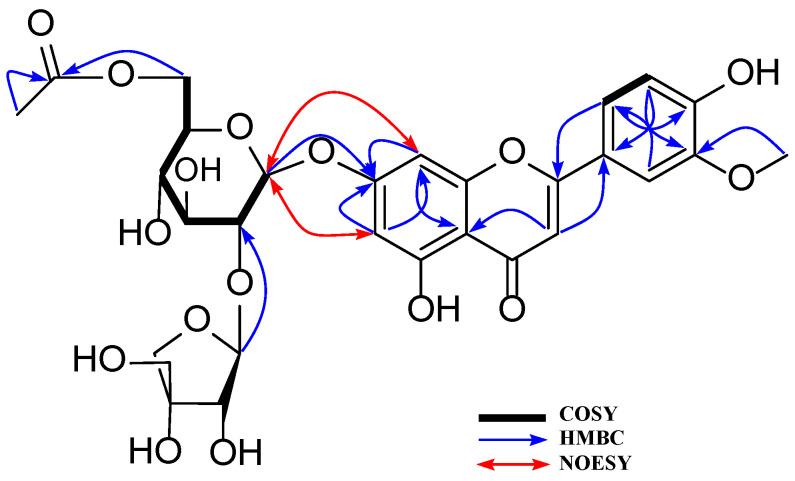
Key COSY, HMBC, and NOESY correlations of compound **7**.

Compounds **2**, **4**, and **9** showed similar characteristics to those of luteolin aglycone in **1** in which the deprotonated ion peak [M − H]^−^ at *m*/*z* 447.0917 of **2** indicated that structural formula of **2** is less of an apiopyranosyl unit than that of **1** (Figure 3). Thus, Compound **2** was identified as luteolin 7-*O*-glucoside. The NMR spectra of **4** showed the downfield signals of two oxygenated protons at *δ*_H_ 4.40 (1H, d, *J* = 11.9 Hz, H_2_-6″) and 4.10 (1H, d, *J* = 11.9, 6.9 Hz, H_2_-6″) coupled to a secondary carbon C-6″ (*δ*_C_ 64.2), which were confirmed by the HSQC spectrum. This information suggested an esterification of the Glc-6 position. Indeed, the identification of the ester produced as Glc (C-6)-OCOR was supported by the HMBC cross-peaks of H_2_-6″ at *δ*_H_ 4.10 to C-1⁗ (*δ*_C_ 167.9), and those of OC-CH_2_-CO at *δ*_H_ 3.51 to C-1⁗ (*δ*_C_ 167.9)/C-3⁗ (*δ*_C_ 167.1) revealed the presence of malonyl group. Furthermore, the negative HR-ESI-MS of **4** produced a deprotonated ion peak [M − H]^−^ at *m*/*z* 665.1373 (Table 1), proving its structure of luteolin 7-*O*-(2-apiosyl-6-malonyl)-glucoside (**4**) [24]. Compound **5** showed a similar structure to that of **1** except for a replacement of a methoxy group. Indeed, its precursor fragments [M − H]^−^ at *m*/*z* 593.1493 and [M − H − Glc − Api]^−^ at *m*/*z* 299.0574 proving the above suggestion (Figure 4). Thus, Compound **5** was identified as chrysoeriol 7-*O*-(2-apiosyl)-glucoside [25].

In contrast, Compounds **3**, **6**, and **8** exhibited the same apigenin aglycone with an AABB coupling spin system (H-2′, -6′ and H-3′, -5′) together with an olefinic proton (H-3) and an AX spin (H-6 and H-8). In addition, their fragmentation showed an ion peak at *m*/*z* 269 (Table 1), proving the above suggestion. Briefly, the ^1^H NMR spectrum of **3** showed significant signals at *δ*_H_ 7.96 (2H, d, *J* = 8.7 Hz, H-2′,6′), 6.94 (2H, d, *J* = 8.7 Hz, H-3′, 5′), 6.87 (1H, s, H-3), 6.81 (1H, d, *J* = 2.1 Hz, H-8), and 6.43 (1H, d, *J* = 2.1 Hz, H-6). Its deprotonated ion peak [M − H]^−^ at *m*/*z* 563.1428 (Table 2) proved the structure of **3** (apiin) [26].

The ^1^H NMR spectrum of Compound **6** displayed an AABB spin system with signals at *δ*_H_ 7.96 (2H, d, *J* = 8.8 Hz, H-2′,6′) and 6.94 (2H, d, *J* = 8.8 Hz, H-3′,5′), which was attributed to the B-ring substituent, whereas an olefinic proton at *δ*_H_ 6.87 (1H, s, H-3) and an AX spin system at *δ*_H_ 6.78 (1H, d, *J* = 2.0 Hz, H-8), 6.42 (1H, d, *J* = 2.0 Hz, H-6) together with two anomeric protons at *δ*_H_ 5.35 (1H, d, *J* = 2.0 Hz, H-1‴), 5.21 (1H, d, *J* = 7.2 Hz, H-1″) were also observed. Its precursor [M − H]^−^ at *m*/*z* 605.1528, identified the structure of 6″-acetylapiin [27].

The ^1^H NMR spectrum of Compound **8** showed an AABB spin system at *δ*_H_ 7.70 (2H, d, *J* = 8.9 Hz, H-2′, 6′), 6.88 (1H d, *J* = 8.9 Hz, H-3′, 5′), and an AX spin system at *δ*_H_ 6.61 (1H, d, *J* = 2.0 Hz, H-8), 6.38 (1H, d, *J* = 2.0 Hz, H-6), and an olefinic proton at *δ*_H_ 6.31 (1H, s, H-3) together two anomeric protons at *δ*_H_ 5.50 (1H, brs, H-1‴), 5.18 (1H, d, *J* = 7.4 Hz, H-1″). Furthermore, an feruloyl moiety was also observed by a *trans*-coupling system at *δ*_H_ 7.27 (1H, d, *J* = 15.9, H-*β*) and 6.01 (1H, d, *J* = 15.9 Hz, H-α), an ABX spin system at *δ*_H_ 6.84 (1H, d, *J* = 1.9 Hz, H-2⁗), 6.73 (1H, dd, *J* = 2.0, 8.2 Hz, H-6⁗), 6.60 (1H, d, *J* = 8.2 Hz, H-5⁗), and a methoxy group at *δ*_H_ 3.74 (3H, s, 3⁗-OCH_3_) together with a peak reduction from [M − H − C_10_H_9_O_3_]^−^ at *m*/*z* 563.1420 (Table 2). Thus, the structure of **8** was determined as apigenin-7-O-[2″-*O*-(5‴-*O*-feruloyl)-D-apiofuranosyl]-D-glucopyranoside [28].

The ^1^H NMR spectrum of **7** displayed the important signals of an ABX spin coupling system with signals at *δ*_H_ 7.56 (1H, d, *J* = 2.1 Hz, H-2′), 7.55 (1H, dd, *J* = 8.0 Hz, 2.1, H-6′), 7.05 (1H, d, *J* = 8.0 Hz, H-5′), which implied the existence of protons of the B-ring substituents. An AX spin coupling system at *δ*_H_ 6.60 (1H, d, *J* = 2.0 Hz, H-8) and 6.39 (1H, d, *J* = 2.0 Hz, H-6) and an olefinic proton at *δ*_H_ 6.84 (1H, s, H-3) were attributed to an A-ring of a flavonoid backbone. Furthermore, two anomeric protons at *δ*_H_ 5.38 (1H, d, *J* = 1.7 Hz, H-1‴) and 5.17 (1H, d, *J* = 7.5 Hz, H-1″), a methoxy group at *δ*_H_ 3.85 (3H, s, 5′-OCH_3_), and a methyl signal at *δ*_H_ 2.02. (3H, s, 6″-CO-CH_3_) were also observed. Its ^13^C NMR spectrum showed the presence of 15 carbons corresponding to a flavonoid backbone, a glucopyranoside, an apiopyranoside moiety, an acetal group with signals at *δ*_C_ 167.9 (6″-CO), a methyl signal at *δ*_C_ 20.3 (6″-CO-CH_3_), and a methoxy group at *δ*_C_ 55.7 (5′-OCH_3_). HMBC long-rang correlations of H-1‴ (*δ*_H_ 5.38) to *δ*_C_ 75.6 (C-2″) and those of CH_3_ (*δ*_H_ 2.02)/H_2_-6″ (*δ*_H_ 4.04) to *δ*_C_ 167.9 (6″-CO) indicated the apiopyranosyl moiety and acetal group linked to the glucopyranoside moiety through C-2″ and C-6″ positions, respectively. In addition, the HMBC spectrum showed the correlations of H-6 (*δ*_H_ 6.39)/H-8 (*δ*_H_ 6.60)/H-1″ (*δ*_H_ 5.17) to *δ*_C_ 162.6 (C-7), revealing the C-7-*O*-glc linkage (Figure 4). The spectroscopic data of Compound **7** were compared to those reported in the literature [23]. Finally, Compound **7** was identified as chrysoeriol 7-*O*-(2-apiosyl-6-acetyl)-glucoside.

The NMR data of **9** are similar to those of **8** except for the presence of an OH-group, identified by its deprotonated ion peak [M − H]^−^ at *m*/*z* 755.1857. The ^1^H NMR spectrum of **9** also exhibited an asymmetric flavone structure of luteolin aglycone with two ABX spin system at *δ*_H_ 7.25 (1H, dd, *J* = 8.2, 2.1 Hz, H-6′), 6.86 (1H d, *J* = 8.2 Hz, H-5′), 6.84 (1H, d, *J* = 1.8 Hz, H-2⁗), 6.75 (1H, dd, *J* = 8.0, 1.8 Hz, H-6⁗), 6.64 (1H, d, *J* = 8.1 Hz, H-5⁗), an AX spin coupling system at *δ*_H_ 6.60 (1H, d, *J* = 2.1 Hz, H-8), 6.40 (1H, d, *J* = 2.1 Hz, H-6), and an olefinic proton at *δ*_H_ 6.29 (1H, s, H-3). Therefore, the structure of **9** was determined as luteolin-[2″-*O*-(5‴-*O*-feruloyl)-D-apiofuranosyl]-D-glucopyranoside by comparison to the data reported in reference [29].

### 3.3. Anti-Inflammatory and Cytotoxic Capacities

**Figure 5:**
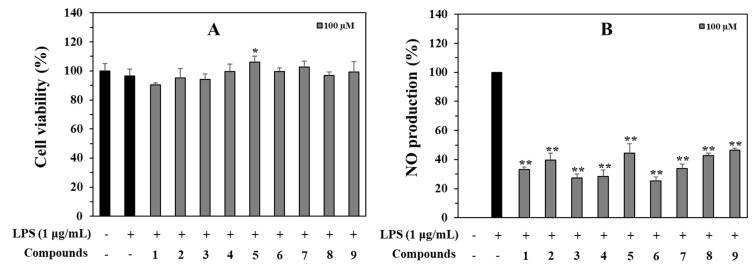
Cytotoxic (**A**) and NO production inhibitory (**B**) effects of isolated compounds (**1**–**9**, 100 μM) in LPS-stimulated RAW264.7 cells. Both experiments were performed in triplicate. The data are represented as mean ± SD. * *p* < 0.05, ** *p* < 0.01, compared to LPS-treated group.

To find the beneficial compounds that have anti-inflammatory properties, the inhibition of NO production was examined in LPS-stimulated RAW264.7 cells by the isolated compounds (**1**–**9**). At the tested concentration of 100 μM, Compounds **3**, **4**, and **6** strongly reduced NO production by 72.7, 71.6 and 74.8% compared to the LPS-treated control, respectively, and Compounds **1**, **2**, **5**, and **7**–**9** displayed a significant inhibition of LPS-induced NO production by 66.7 to 53.5% at the same tested concentration. None of these isolates were cytotoxic (Figure 5).

### 3.4. Inhibitory Mechanism of Flavonoid Glycosides against iNOS and COX-2 Levels Expression

**Figure 6:**
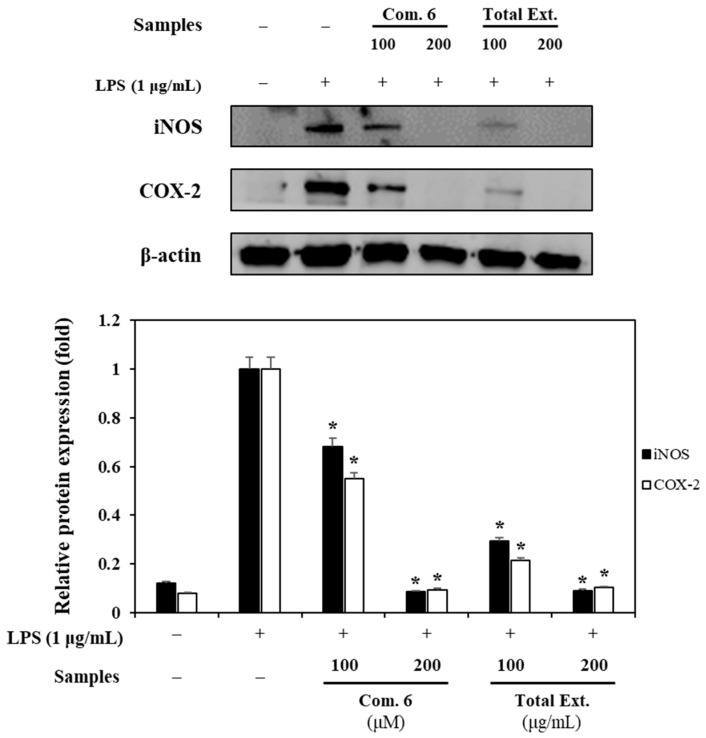
Effects of Compound **6** and total extract (Total Ext.) on iNOS and COX-2 expressions induced by LPS on RAW264.7 cells. Relative density was calculated as the ratio of the expression level of each protein with β-actin. The data are expressed as the mean ± SD (*n* = 3). * *p* < 0.01, compared with the LPS-stimulated group.

**Figure 7:**
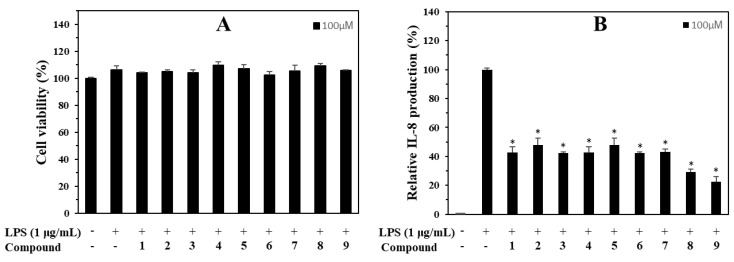
Cytotoxic (**A**) and IL-8 production inhibitory (**B**) effects of compounds in LPS-induced HT-29 cells. HT-29 cells were treated with compounds **1**−**9** (100 µM) for 2 h and stimulated with LPS (100 ng/mL) for 12 h. (**A**) The viability of cells was determined using an MTT assay. (**B**) The level of IL-8 in the culture media was measured with an ELISA kit. The values are expressed as mean ± standard deviation of three individual experiments. * *p* < 0.01, compared to the LPS-treated group.

The action modes of the most active compound (**6**) were further examined for both enzymatic proteins. A Western blotting assay was, therefore, conducted to elucidate the potential mechanism. The inhibition of the iNOS and COX-2 expressions by the strongest active compound (**6**) and the total extract were investigated (Figure 6). Compound **6** and the total extract markedly reduced the expression levels of both inflammatory genes in RAW264.7 cells compared to those of *β*-actin in LPS-pre-treated cells without added sample (Figure 7). The result suggested that these inhibitors may affect NO production by inhibiting catalyst enzymes iNOS and COX-2 in the rate-limiting steps to produce NO and PGE2, respectively.

### 3.5. Anti-Inflammatory Capacity on IL-8 Production in LPS-Stimulated HT-29 Cells

In addition, all the isolates (**1**–**9**) were evaluated for their ability to inhibit IL-8 production. Compared to the LPS-treated control without sample addition, Compound **9** exhibited the strongest activity of LPS-induced IL-8 production in HT-29 cells, with an inhibition rate of 77.4% at 100 μM. Compound **8** showed a significant inhibition rate of 70.9%, followed by other compounds (**1**–**7**) with inhibition rates ranging from 57.3 to 52.1% compared to LPS-induced IL-8 production without sample pre-treatment at the same tested concentration of 100 μM. In contrast, no tested samples showed any significant cytotoxicity in HT-29 cells at the tested conditions by the MTT assay (Figure 7).

### 3.6. Molecular Docking Analysis

**Figure 8:**
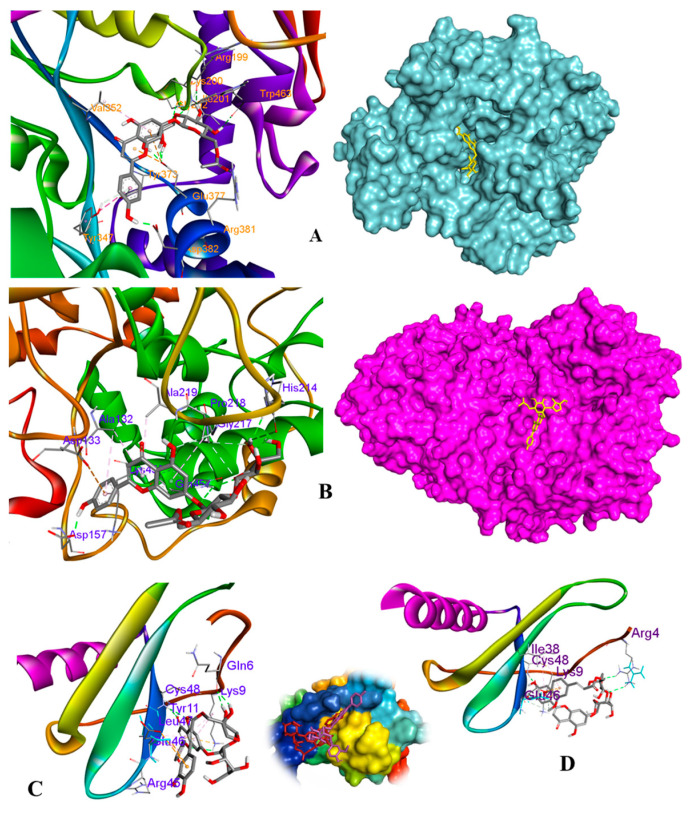
Three-dimensional view and interacting residues in the 3E7G–Compound **6** (**A**), 5IKQ–Compound **6** (**B**), 5D14–Compound **8** (**C**), and 5D14–Compound **9** (**D**) complexes.

Molecular docking simulation was applied to compute the binding affinity and interactions between receptors and Compounds **6**, **8**, and **9** in silico discovery (Figures S33–S35). The result showed that the iNOS–Compound **6** complex exhibited a strong docking energy of −9.50 kcal/mol. This may be explained by the interactions between Compound **6** and the active residues of iNOS protein (Figure 8, Table 3).

**Figure 9:**
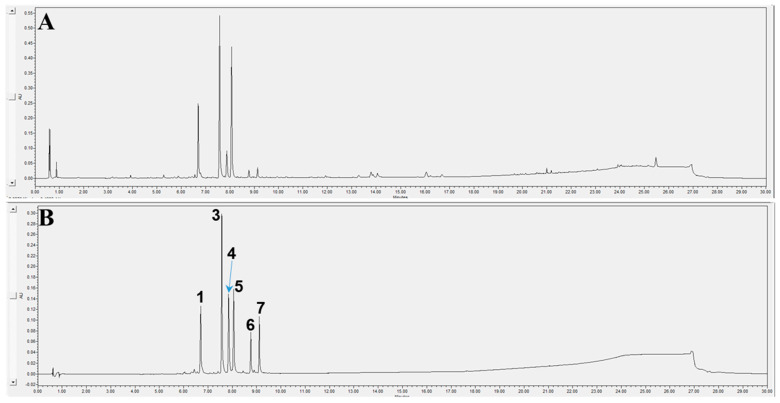
UPLC chromatograms of total extract (**A**) and six marker compounds (**B**) obtained from the herbal extract of *V*. *tetrasperma* detected at 254 nm.

In detail, the ligand was stabilized by the formation of **7** conventional hydrogen bonds with amino acids, including ARG199, ARG381, ASP382, ILE201, GLU377, GLY202, and TRO463 along with a hydrophobic interaction at VAL352. The above observation suggested that the docked ligand occupied the binding pocket of the iNOS structure. Moreover, the iNOS–compound complex showed additional interactions (Table 2) with active residues in the binding pocket of the iNOS protein (Figure 9) in which the amino acids GLU377, ARG381, and VAL352 are the important residues of the binding sites in the pocket [30,31,32].

Furthermore, the COX-2–Compound **6** docked complex showed docking energy of −3.28 kcal/mol (Table 3) along with the formation of three hydrogen bonds at ASP157, GLN457, and HIS214 (Figure 8). Moreover, all the ligands exhibited additional interactions including hydrophobic, van der Waals, and polar interactions with active residues surrounding them in the binding pocket of the protein.

As shown in Table 3, Compound **9** displayed a lower binding affinity (−7.75 kcal/mol) than that (−4.32 kcal/mol) of Compound **8** when it was docked to the 5D14 (IL-8) receptor. This observation is associated with their strong inhibition on IL-8 production as seen in the above experimental data. Importantly, these ligands showed interactions with amino acids GLN6, LYS9, and CYS48 surrounding Compound **8** and those of ARG4 and CYS48 bounding Compound **9** through conventional hydrogen bonds, respectively, at the binding pockets of established complexes. On the other hand, these ligands also displayed interactions with other active residues of the IL-8 protein.

Thus, these docking analyses support the data approach of the above Western blotting and IL-8 experimental results.

### 3.7. Establishment and Validation Analysis of Marker Compounds (1 and 3–7) from the Herbal Extract of V. teterasperma

An analytical method was established and applied to build a chromatogram of the *V*. *teterasperma* extract. The chromatographic profile was obtained by optimizing the analytical factors, including the mobile phase, gradient elution, column, wavelength detection, and flow rate, as well as resolution for peak separation (Figure 9).

Linearity regression, LODs, and LOQs: linearity is the capability to obtain data that are proportional to the concentration of the analyte in the sample within a given range. It was calculated between peak areas and concentrations, and was performed using six calibration curves built at different concentrations of the standard solutions of each analyte, with the correlation coefficients (*r*^2^) ranging from 0.9990 to 0.9997. These data indicated that the responses of the standards in the working ranges of the concentrations were linear and satisfied. The LOD and LOQ values, which expressed the sensitivity of the system, were calculated, and ranged from 0.12 to 0.60 μg/mL and from 0.39 to 1.84 μg/mL, respectively (Table 4).

### 3.8. Qualification of Marker Compounds from the V. tetrasperma Extract

The analytical method aimed to qualify the content of an individual marker compound in the total extract of the plant. The above established method was applied to determine the average content of each marker compound in the total extract of *V*. *tetrasperma*. Compound **3** had the highest content of 3.02 mg/g. Compounds **1** and **5** displayed medium amounts of 2.18 and 1.94 mg/g, respectively. Compound **4** had a content of 0.39 mg/g. Compounds **6** and **7** exhibited similar contents at 0.09 and 0.10 mg/g, respectively (Table 4).

## 4. Discussion

The antioxidant and anti-inflammatory effects of the total extract and fractions derived from *V*. *teterasperma* guided the separation and identification of nine compounds from the active (EtOAc) fraction. The structures of isolated compounds (**1**–**9**) were established by using spectroscopic techniques and compared to those reported in the literatures. Among the isolates, chrysoeriol 7-*O*-(2-apiosyl-6-acetyl)-glucoside (**7**) was reported for the first time in the NMR data of the current study. Other isolates, such as luteolin 7-*O*-[2-*β*-D-apiofuranosyl)-*β*-D-glucopyranoside (**1**), luteolin 7-*O*-glucoside (**2**), apiin (**3**), luteolin 7-*O*-(2-apiosyl-6-malonyl)glucoside (**4**), chrysoeriol 7-*O*-(2-apiosyl)glucoside (**5**), 6″-acetylapiin (**6**), apigenin-7-*O*-[2″-*O*-(5‴-*O*-feruloyl)-D-apiofuranosyl]-D-glucopyranoside (**8**), and luteolin-[2″-*O*-(5‴-*O*-feruloyl)-D-apiofuranosyl]-D-glucopyranoside (**9**), were isolated for the first time from this plant. These isolated compounds displayed a significant inhibition on NO production in LPS-stimulated RAW264.7 cells with different inhibition rates at the tested concentration of 100 μM. Based on the NO-producing activity of the isolates and compounds (**3**, **5**, **6**, **7**, and **8**) could be distinguished by 4′-OCH_3_ and 6‴-feruloyl-functional groups. However, Compound **6** exhibited the strongest inhibitory effect on NO production derived from LPS-mediated RAW264.7 cells at the same concentration, and Compound **8** exhibited the weakest inhibition of NO production. The results indicated that the 4′-OCH_3_ and 6‴-feruloyl-functional groups were not favored by these flavonoid glycoside compounds. Compounds **1**, **2**, **4**, and **9** displayed the same luteolin-glucoside derivatives. However, Compound **4** exhibited stronger NO inhibition than Compound **1**, followed by Compounds **2** and **9**. Therefore, malonyl and apiosyl moieties were important in promoting the inhibition of NO production under the tested conditions. Our study is the first to report the inhibitory effect of Compounds **1** and **4**–**7** on LPS-induced NO production in RAW264.7 cells.

The strong active compound 6″-acetylapiin exhibited a reduction in the levels of expression of both catalyst enzymes, iNOS and COX-2. The result suggested that these inhibitors may affect NO production by inhibiting catalyst enzymes iNOS and COX-2 in the rate-limiting steps to produce NO and PGE2, respectively. 6″-Acetylapiin first expressed the inhibitory mechanism of suppressing (iNOS and COX-2) isoform expression in this study. Previously, Active Compounds **3** and **4** had been reported to have antioxidant [23,33] and anti-inflammatory [34,35] effects, as well as an anti-hyperuricemic effect by reducing the uric-acid-lowering effects in in vivo [36] studies.

In addition, the isolated compounds (**1**–**9**) also inhibited IL-8 production in LPS stimulated HT-29 cells with different inhibition rates. A structural activity relationship indicated that the key functional group inhibiting IL-8 production was correlated with the presence of a feruloyl moiety in the structures of the compounds (**8** and **9**). Thus, this functional group was important in the inhibition of LPS-induced IL-8 production in HT-29 cells. This is the first report of the inhibitory effect of Compounds **1** and **4**–**7** on LPS-induced IL-8 production in HT-29 cells.

Our current study is not only the first to report a chemical investigation of the above flavonoid glycosides, but also the first to report the antioxidant and anti-inflammatory properties of this plant. Furthermore, this study reported for the first time that 6″-acetylapiin’s anti-inflammatory actions regulate NO, iNOS, and COX-2 production in LPS-stimulated RAW264.7 cells.

An in silico approach further confirmed this inhibitory effects by evaluating the binding affinities of the Active Compounds **6**, **8** and **9** to iNOS, COX-2, and IL-8 proteins. The docking output result was associated with the experimental result indicating the bioactivities of isolates (**6**, **8**, and **9**). In detail, Compound **6** docked to both iNOS and COX-2 receptors with low binding energies (−9.50 and −3.28 Kcal/mol, respectively) surrounded by hydrogen and hydrophobic interactions (Table 2) in the pockets of ligand–receptor complexes (Figure 9). In addition, Compounds **8** and **9** occurred in the same pocket of the IL-8 (PDB ID: 5D14) receptor (Figure 8) and interacted with key amino acids (ARG4, GLN6, and CYS48) at the binding pockets [30].

An analytical method was successfully established with sensitivity (LODs and LOQs ranging from 0.12 to 0.60 μg/mL and from 0.39 to 1.84 μg/mL, respectively), showing the resolution of peak separations from each of the six maker compounds presented in the total extract of this plant in the chromatogram. The appreciate values (0.9990 ≤ *r*^2^ ≤ 0.9997) of correlation coefficients also displayed the linearity regression. This established method was then employed to quantitate the marker content from the extract. As a result, the active compounds, Compounds **1** and **3,** expressed high levels with amounts of 2.18 and 3.02 mg/g, respectively, reasonably considered as important components promoting the anti-inflammatory capacity of the extract of this plant. On the other hand, the content of other active components, Compounds **4** and **6**, with amounts of 0.39 and 0.09 mg/g, respectively, may contribute to the enhancement of the anti-inflammatory property of this plant.

Further studies should be performed with a large amount of dried material to enhance the study of the biomass of active compounds found in this primary study which provides support for in vivo studies in the future.

## 5. Conclusions

In this study, we firstly reported antioxidant and anti-inflammatory activities through DPPH/ABTS radical scavenging activities, as well as inhibitory effects against ROS, and NO production, respectively. The current study also revealed the separation of flavonoid glycosides obtained from *V*. *tetrasperma* for the first time, which resulted in the identification of nine compounds. Among them, chrysoeriol 7-*O*-(2-apiosyl-6-acetyl)-glucoside was reported for the first time in NMR data. This is also the first report on the antioxidant and anti-inflammatory properties of this herbal food by inhibiting NO and IL-8 production in RAW264.7 and HT-29 cells, respectively. We firstly reported on the inhibitory effects of Compounds **1** and **4**–**7** in LPS-induced NO production on RAW264.7 cells and in LPS-induced IL-8 production on HT-29 cells, respectively. Further experiments with the strongly active compound were conducted to assess the inhibitory mechanism of suppressing (iNOS and COX-2) isoform expression for the first time. The active compounds with anti-inflammatory effects and their content in the herbal extract clarified the inhibitory capacity of *V*. *tetrasperma*. The molecular docking analysis further proved the anti-inflammatory mode of action by inhibiting the enzymatic activities of iNOS, COX-2, and of the IL-8 protein during the reaction process to produce mediators and cytokine production. The interesting activities this study has found indicate the new value of *V*. *tetrasperma*, which is consumed as a vegetable daily, and offered some evidence for the usage and development of this plant in the field of functional food to guide the treatment of inflammatory diseases in the future. This study also provided an effective tool for assessing the quality of this herbal food on the market through a UPLC analytical method using the above six standards. Our findings highlight the significance of this herb as a potential source of previously unrecognized health-promoting substances.

## Figures and Tables

**Figure 2:**
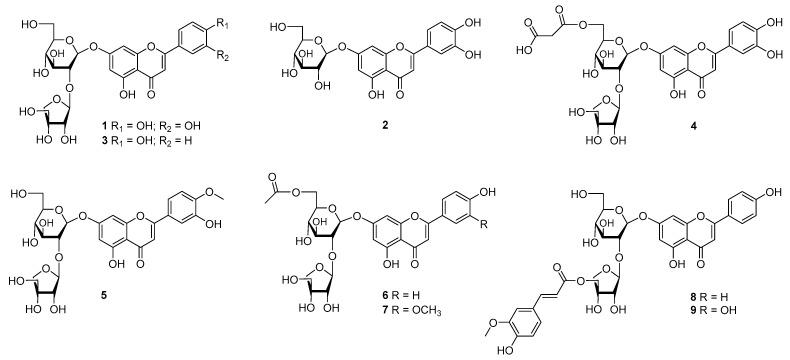
Chemical structures of constituents isolated from *V*. *tetrasperma*.

**Table 1:** Antioxidant activity of total extract and fractions of *V*. *tetrasperma*.

Sample	Antioxidant Activity (μmol TEAC/g Sample)	
	DPPH	ABTS
Total extract	0.089 ± 0.011	0.186 ± 0.013
*n*-Hexane	0.131 ± 0.013	0.020 ± 0.001
CH_2_Cl_2_	0.152 ± 0.011	0.254 ± 0.008
EtOAc	0.281 ± 0.015	0.482 ± 0.025
*n*-BuOH	0.172 ± 0.016	0.226 ± 0.018
H_2_O	0.077 ± 0.011	0.090 ± 0.003

Antioxidant capacity were expressed as mean ± SD (*n* = 3).

**Table 2:** MS/MS fragmentations of compounds **1**–**9**.

Compounds	Formula	Molecular Weight	Adduct	Found	Error	MS/MS Product Ions
				(*m*/*z*)	(ppm)	
Luteolin 7-*O*-[2-*β*-D-apiofuranosyl)-*β*-D-glucopyranoside **(1)**	C_26_H_28_O_15_	580.1428	[M − H]^−^	579.1371	1.6	579.1371, 447.0919, 285.0403
Luteolin 7-*O*-glucoside **(2)**	C_21_H_20_O_11_	448.1006	[M − H]^−^	447.0917	−2.9	447.0917, 286.0432
Apiin **(3)**	C_26_H_28_O_14_	564.14790	[M − H]^−^	563.1428	2.7	563.1428, 431.0976, 269.0469
Luteolin 7-*O*-(2-apiosyl-6-malonyl)glucoside **(4)**	C_29_H_30_O_18_	666.1432	[M − H]^−^	665.1373	1.1	665.1373, 621.1457, 489.1041, 285.0403
Chrysoeriol 7-*O*-(2-apiosyl)glucoside **(5)**	C_27_H_30_O_15_	594.1585	[M − H]^−^	593.1509	−2.5	593.1509, 299.0576
6″-Acetylapiin **(6)**	C_28_H_30_O_15_	606.1585	[M − H]^−^	605.1528	2.9	605.1528, 563.1426, 269.0471
Chrysoeriol 7-*O*-(2-apiosyl-6-acetyl)glucoside **(7)**	C_29_H_32_O_16_	636.1690	[M − H]^−^	635.1634	2.8	635.1634, 593.1504, 299.0574
Apigenin-7-*O*-[2″-*O*-(5‴-O-feruloyl)-D-apiofuranosyl]-D-glucopyranoside **(8)**	C_36_H_36_O_17_	740.1952	[M − H]^−^	739.1878	0.4	739.1878, 545.1303, 269.0456
Luteolin-[2″-*O*-(5‴-*O*-feruloyl)-D-apiofuranosyl]-D-glucopyranoside **(9)**	C_36_H_36_O_18_	756.1902	[M − H]^−^	755.1857	2.6	755.1857, 579.1351, 447.0849, 285.0406

**Table 3:** AutoDock calculated affinities for docking of Compounds **6**, **8,** and **9** docked to iNOS, COX-2, and IL-8 proteins.

Ligand	Protein	Affinity Score (Kcal/mol)	Conventional Hydrogen Bond	Hydrophobic Interactions
6″-Acetylapiin (**6**)	PDB ID: 3E7G	−9.50	ARG199, ARG381, ASP382, ILE201, GLU377, GLY202, TRO463	VAL352
6″-Acetylapiin (**6**)	PDB ID: 5IKQ	−3.28	ASP157, GLN457, HIS214	ALA132, ALA219, LYS459
Apigenin-7-*O*-[2″-*O*-(5‴-*O*-feruloyl)-D-apiofuranosyl]-D-glucopyranoside (**8**)	PDB ID: 5D14	−4.32	GLN6, LYS9, CYS48	CYS7, ILE8, THR10, LYS13, TYR11, ILE38, GLU46, ARG45, LEU47
Luteolin-[2″-*O*-(5‴-*O*-feruloyl)-D-apiofuranosyl]-D-glucopyranoside (**9**)	PDB ID: 5D14	−7.75	ARG4, CYS48	LEU3, CYS5, GLN6, CYS7, ILE8, THR10, TYR11, ILE38, GLN46, LEU47

**Table 4:** Contents of six marker compounds (**1**, **3**–**7**) in *V*. *tetrasperma*.

Marker Compound	Concentration Range (µg/mL)	Regression Equation	Correlation Coefficient (*r*^2^)	LOD (µg/mL)	LOQ (µg/mL)	Content (*w*/*w*)
Luteolin 7-*O*-[2-*β*-D-apiofuranosyl)-*β*-D-glucopyranoside **(1)**	1.95~250	Y = 6342.7x + 9788.7	0.9997	0.60	1.84	2.18
Apiin **(3)**	1.95~250	Y = 12,674x + 20,920	0.9997	0.14	0.42	3.02
Luteolin 7-*O*-(2-apiosyl-6-malonylglucoside) **(4)**	0.39~50	Y = 13,457x + 4379.3	0.9994	0.12	0.39	0.39
Chrysoeriol 7-*O*-(2-apiosyl)-glucoside **(5)**	1.95~250	Y = 11,866x + 25,034	0.9997	0.53	1.62	1.94
6″-Acetylapiin **(6)**	0.31~20	Y = 15,520x + 1345.3	0.9995	0.28	0.85	0.09
Chrysoeriol 7-*O*-(2-apiosyl-6-acetyl)-glucoside **(7)**	0.31~20	Y = 18,736x − 2737.6	0.9990	0.52	1.59	0.10

## Data Availability

All of data are contained within the article.

## Supplementary Materials

The following supporting information can be downloaded at: https://www.mdpi.com/article/10.3390/antiox12051044/s1, Figures S1–S32: spectroscopic spectra of compounds **1**–**9**; Figure S33: Interactions between binding sites of iNOS and compound **6**; Figure S34: Interactions between binding sites of COX-2 and compound **6**; Figure S35: Interactions between binding sites of the IL-8 receptor and compounds (**8** and **9**).
