# Supplementary material for: Antioxidant and Anti-Inflammatory Capacities of Fractions and Constituents from Vicia tetrasperma
Source: Antioxidants (Basel). 2023 May 4;12(5):1044. doi: 10.3390/antiox12051044 (PMC10215666; doi:10.3390/antiox12051044)
Supplement: Supplementary file 1 [file antioxidants-12-01044-s001.zip › antioxidants-2330661-supplementary.pdf]

Supplementary materials

Antioxidant and anti-inflammatory capacities of fractions and constituents from *Vicia tetrasperma*

Duc Dat Le[1,†], Kyung Hyun Min[2,†] and Mina Lee[1,*]

[1] College of Pharmacy, Research Institute of Life and Pharmaceutical Sciences, Sunchon National University, 255 Jungangno, Suncheon 57922, Jeonnam, Republic of Korea; ddle@scnu.ac.kr
[2] School of Pharmacy and Institute of New Drug Development, Jeonbuk National University, Jeonju 54896, Republic of Korea; khmin1492@jbnu.ac.kr
* Correspondence: minalee@sunchon.ac.kr or minalee@scnu.ac.kr; Tel.: +82-61-750-3764; Fax: +82-61-750-3708
† These authors contributed equally to this work.

Table of Contents

*1. Spectroscopic and spectrometry spectra of compounds (**1**–**9**)* 1

Figure S1. ^{1}H NMR spectrum of compound **1**....................1

Figure S2. ^{13}C NMR spectrum of compound **1**....................1

Figure S3. ^{1}H-^{13}C HSQC spectrum of compound **1**....................2

Figure S4. ^{1}H-^{13}C HMBC spectrum of compound **1** 2

Figure S5. HR-ESI-MS/MS spectrum of compound **1** 3

Figure S6. ^{1}H NMR spectrum of compound **2**....................3

Figure S7. ^{13}C NMR spectrum of compound **2**....................3

Figure S8. HR-ESI-MS/MS spectrum of compound **2** 4

Figure S9. ^{1}H NMR spectrum of compound **3**....................4

Figure S10. ^{13}C NMR spectrum of compound **3**....................4

Figure S11. HR-ESI-MS/MS spectrum of compound **3** 5

Figure S12. ^{1}H NMR spectrum of compound **4**....................5

Figure S13. ^{13}C NMR spectrum of compound **4**....................5

Figure S14. ^{1}H-^{13}C HSQC spectrum of compound **4**....................6

Figure S15. ^{1}H-^{13}C HMBC spectrum of compound **4** 6

Figure S16. HR-ESI-MS/MS spectrum of compound **4** 7

Figure S17. ^{1}H NMR spectrum of compound **5**....................7

Figure S18. ^{13}C NMR spectrum of compound **5**....7

Figure S19. HR-ESI-MS/MS spectrum of compound **5**....8

Figure S20. ^{1}H NMR spectrum of compound **6**....8

Figure S21. ^{13}C NMR spectrum of compound **6**....8

Figure S22. HR-ESI-MS/MS spectrum of compound **6**....9

Figure S23. ^{1}H NMR spectrum of compound **7**....9

Figure S24. ^{13}C NMR spectrum of compound **7**....9

Figure S25. ^{1}H-^{13}C HSQC spectrum of compound **7**....10

Figure S26. ^{1}H-^{13}C HMBC spectrum of compound **7**....10

Figure S27. HR-ESI-MS/MS spectrum of compound **7**....11

Figure S28. ^{1}H NMR spectrum of compound **8**....11

Figure S29. ^{13}C NMR spectrum of compound **8**....11

Figure S30. HR-ESI-MS/MS spectrum of compound **8**....12

Figure S31. ^{1}H NMR spectrum of compound **9**....12

Figure S32. HR-ESI-MS/MS spectrum of compound **9**....12

2. In silico studies....13

Figure S33. Interactions between binding sites of the iNOS receptor with respect to ligand (compound **6**)....13

Figure S34. Interactions between binding sites of the COX-2 receptor with respect to ligand (compound **6**)....14

Figure S35. Interactions between binding sites of the IL-8 receptor with respect to ligands [compounds **8** (**A**) and **9** (**B**)]....15

*1. Spectroscopic and spectrometry spectra of compounds (**1**–**9**):*

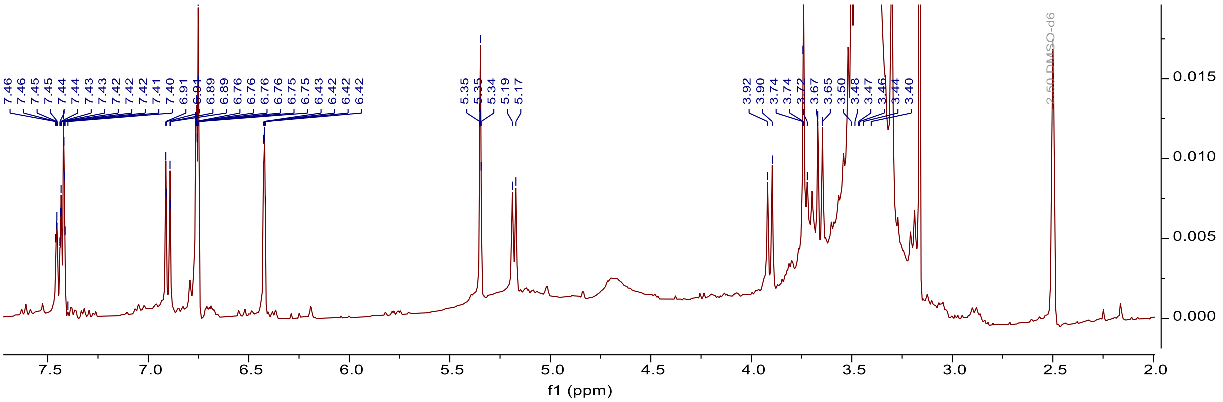

Figure S1. ^{1}H NMR spectrum of compound **1**.

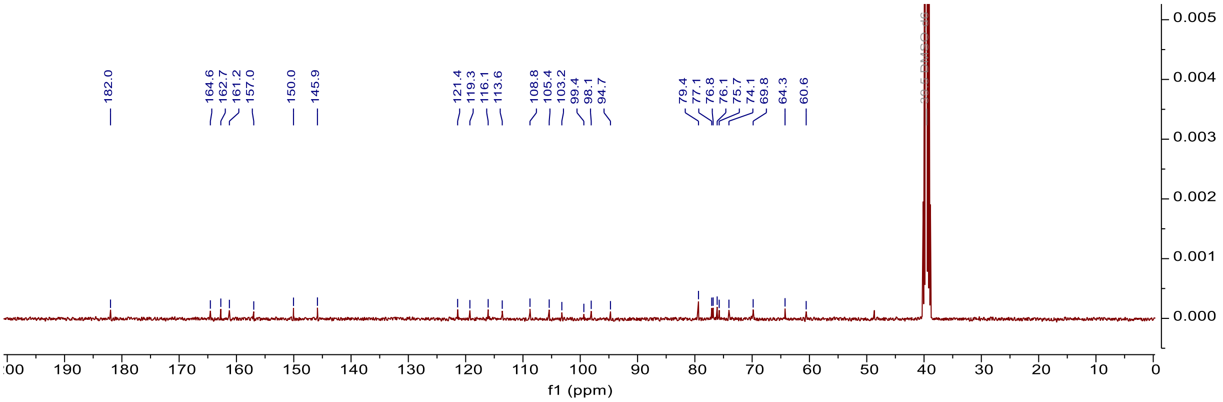

Figure S2. ^{13}C NMR spectrum of compound **1**.

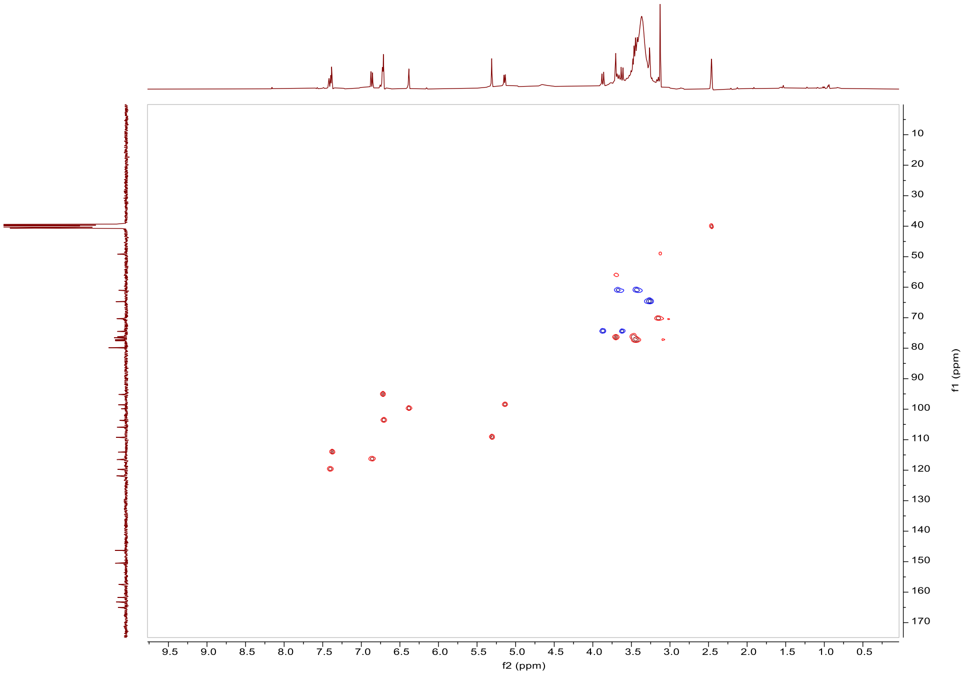

Figure S3. ^{1}H-^{13}C HSQC spectrum of compound **1**.

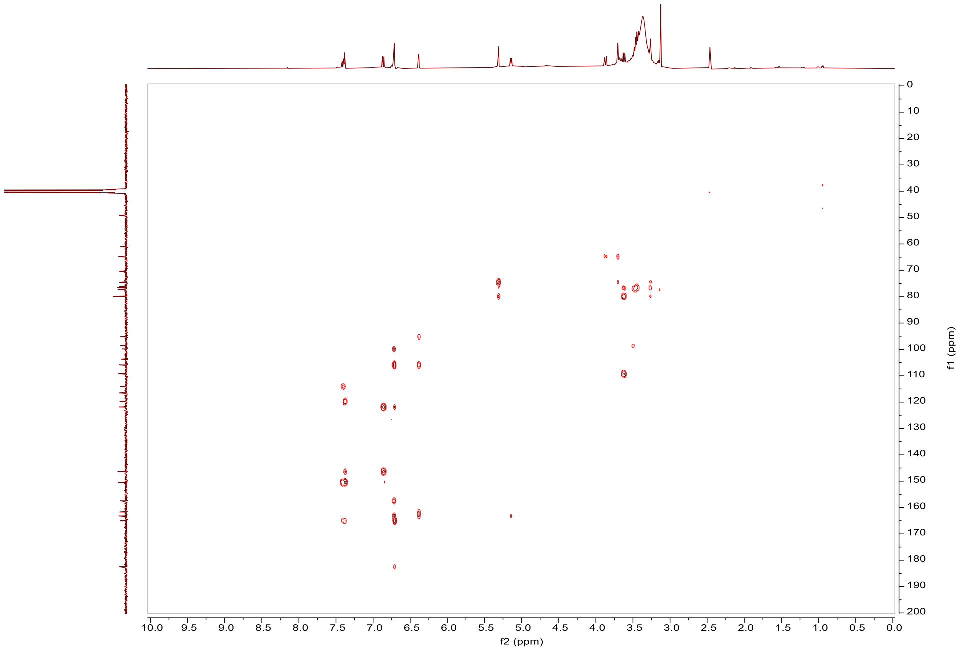

Figure S4. ^{1}H-^{13}C HMBC spectrum of compound **1**.

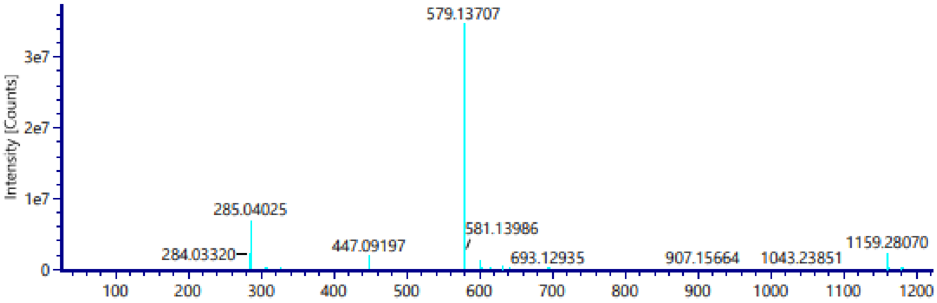

Figure S5. HR-ESI-MS/MS spectrum of compound **1**.

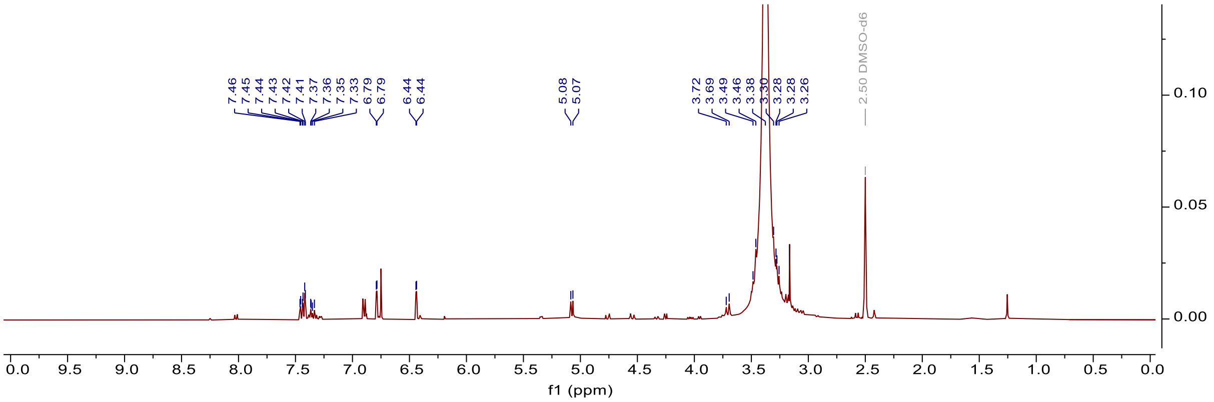

Figure S6. ^{1}H NMR spectrum of compound **2**.

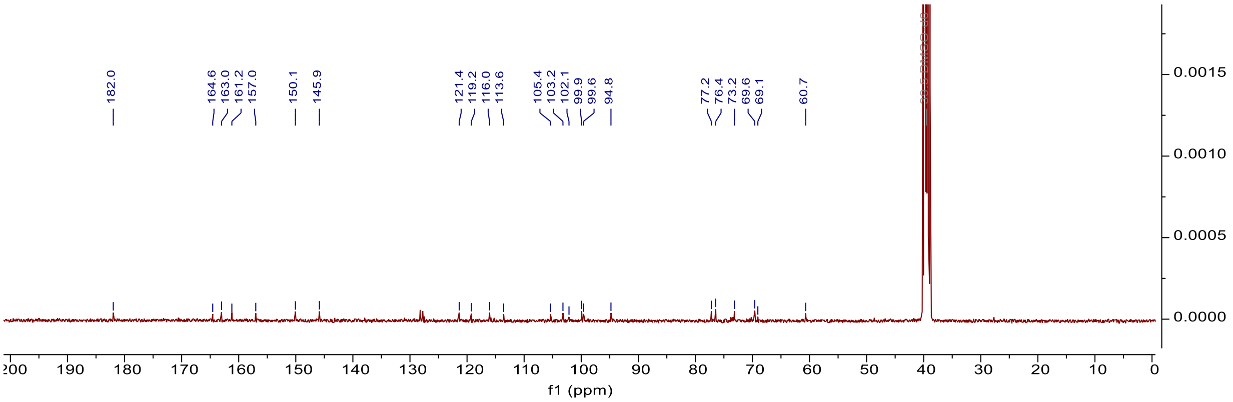

Figure S7. ^{13}C NMR spectrum of compound **2**.

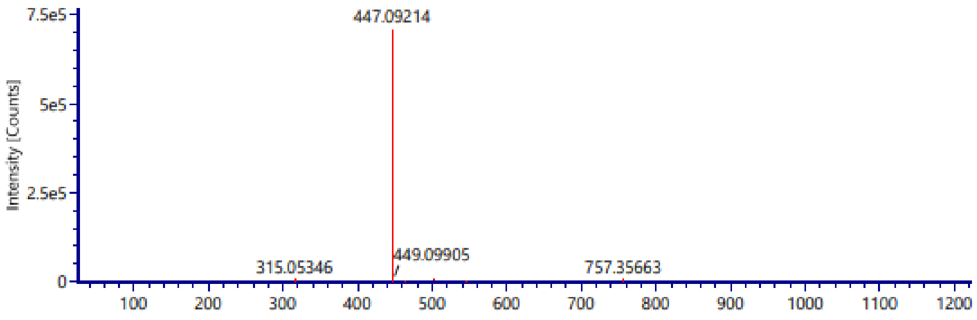

Figure S8. HR-ESI-MS/MS spectrum of compound **2**.

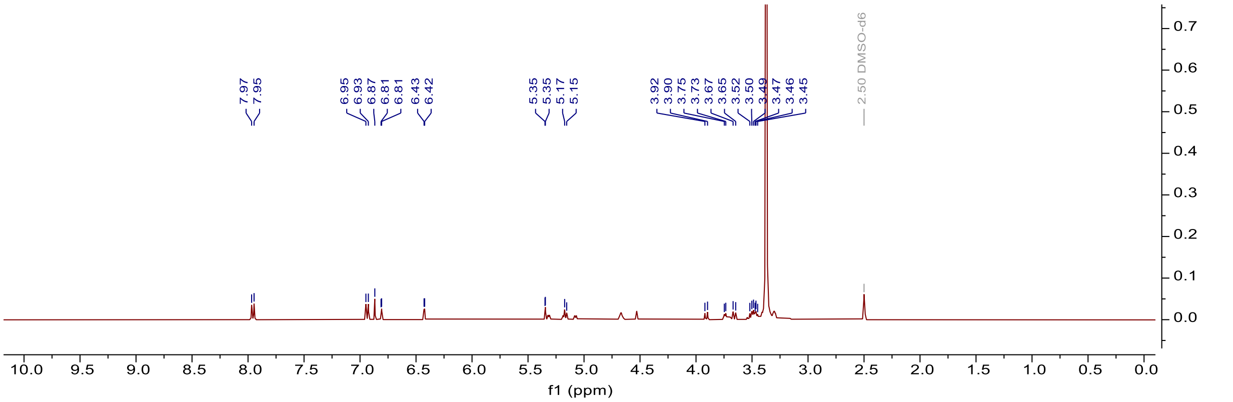

Figure S9. ^{1}H NMR spectrum of compound **3**.

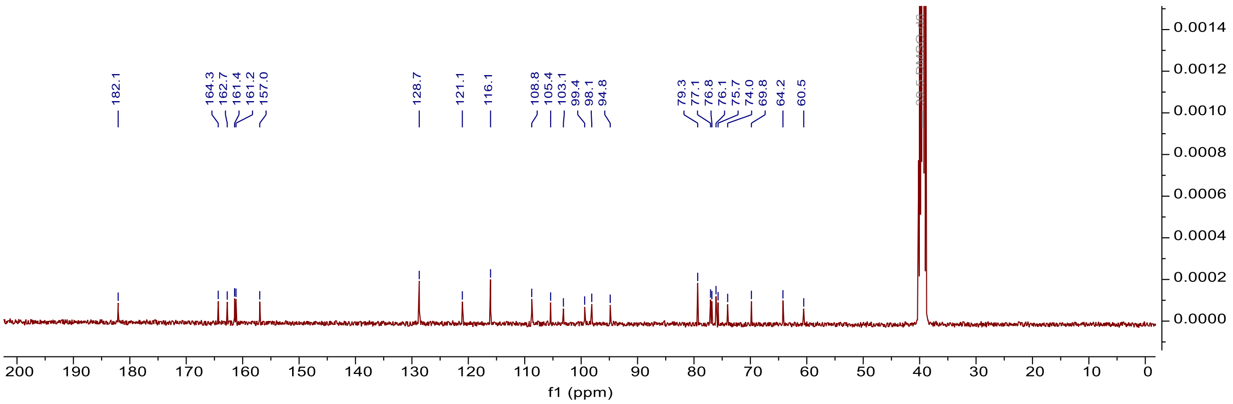

Figure S10. ^{13}C NMR spectrum of compound **3**.

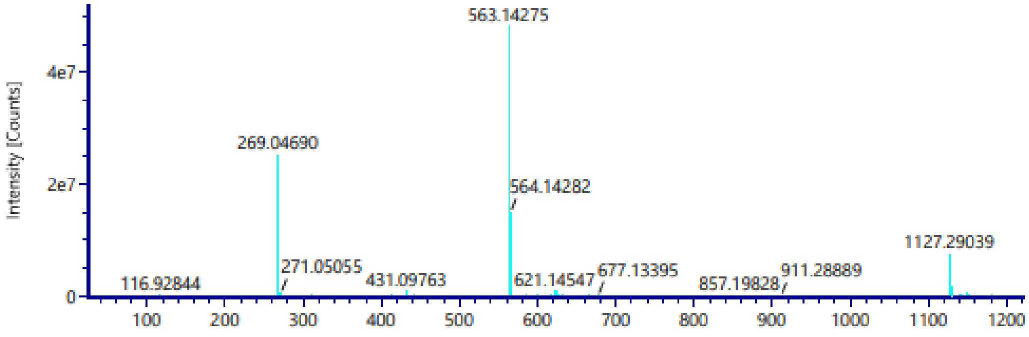

Figure S11. HR-ESI-MS/MS spectrum of compound **3**.

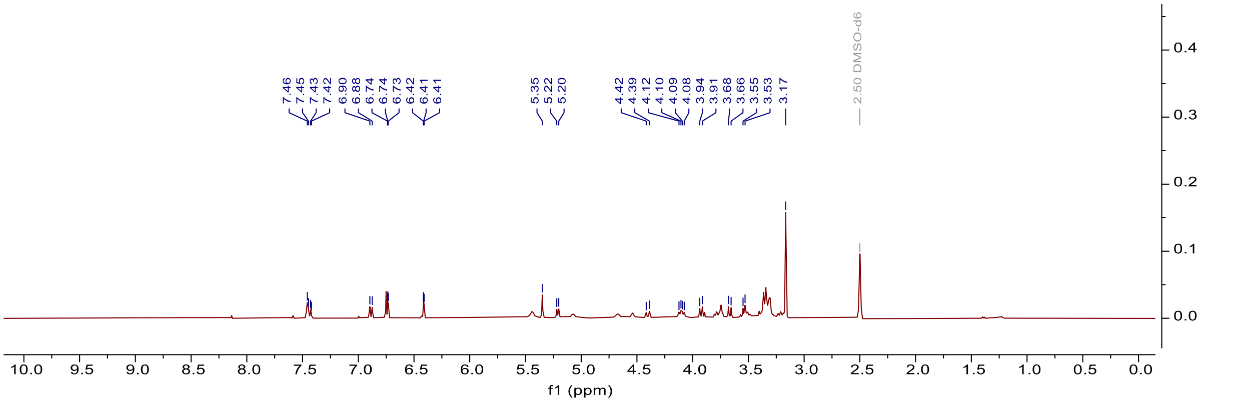

Figure S12. ^{1}H NMR spectrum of compound **4**.

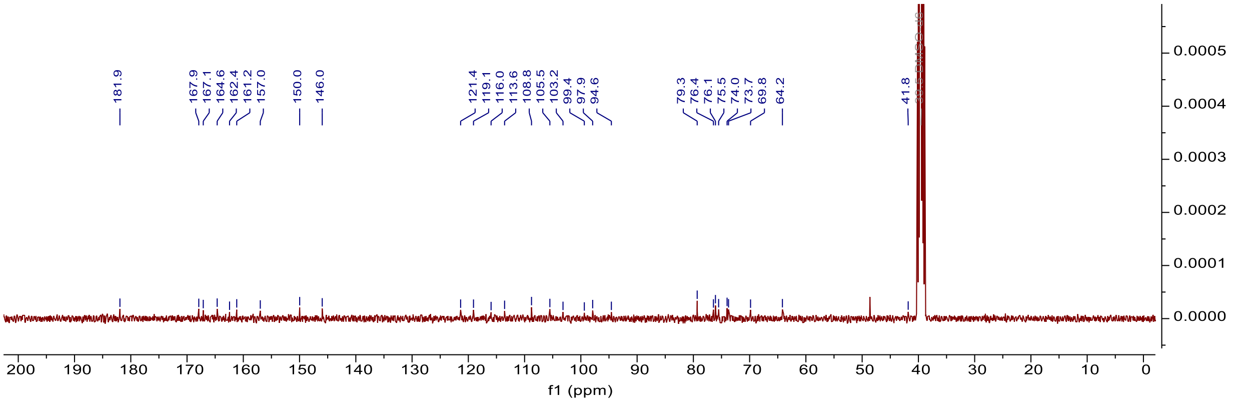

Figure S13. ^{13}C NMR spectrum of compound **4**.

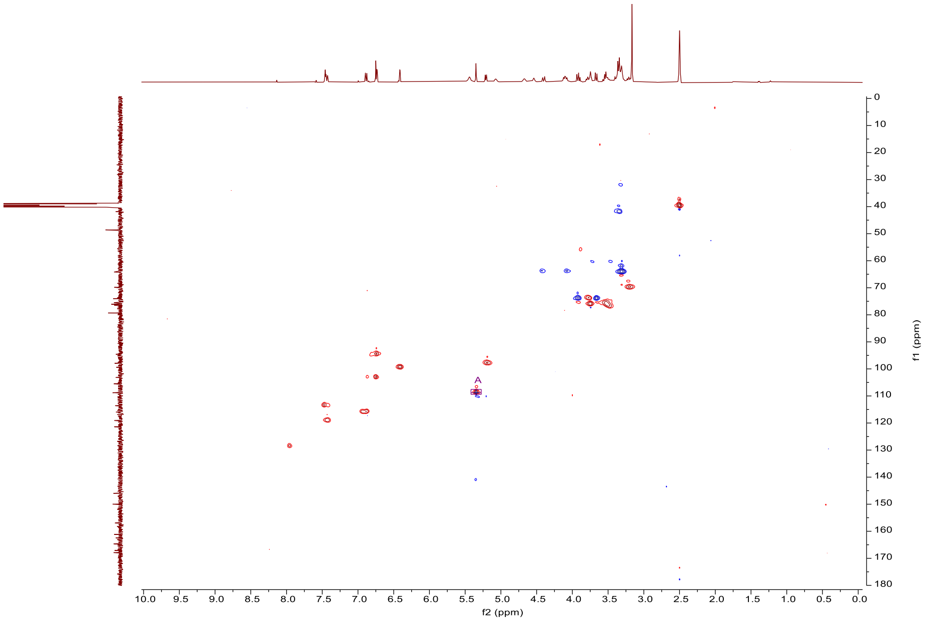

Figure S14. ^{1}H-^{13}C HSQC spectrum of compound **4**.

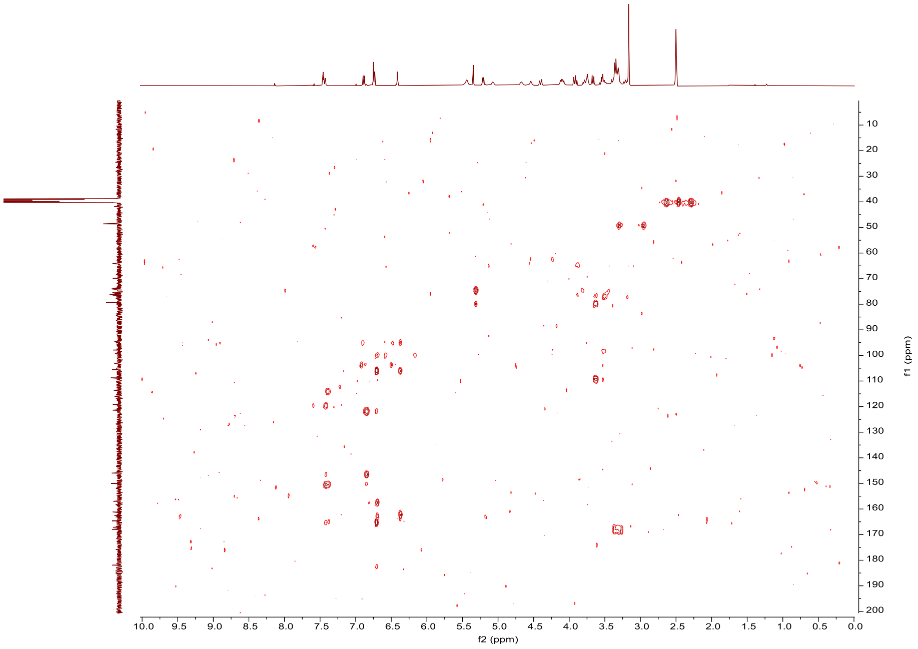

Figure S15. ^{1}H-^{13}C HMBC spectrum of compound **4**.

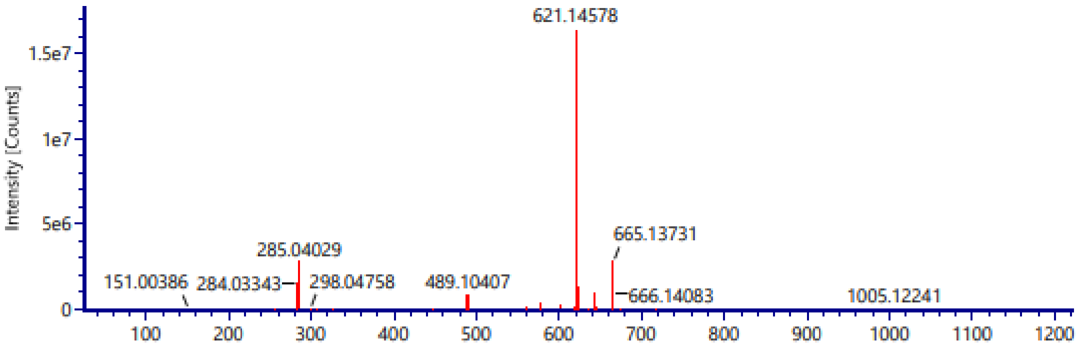

Figure S16. HR-ESI-MS/MS spectrum of compound **4**.

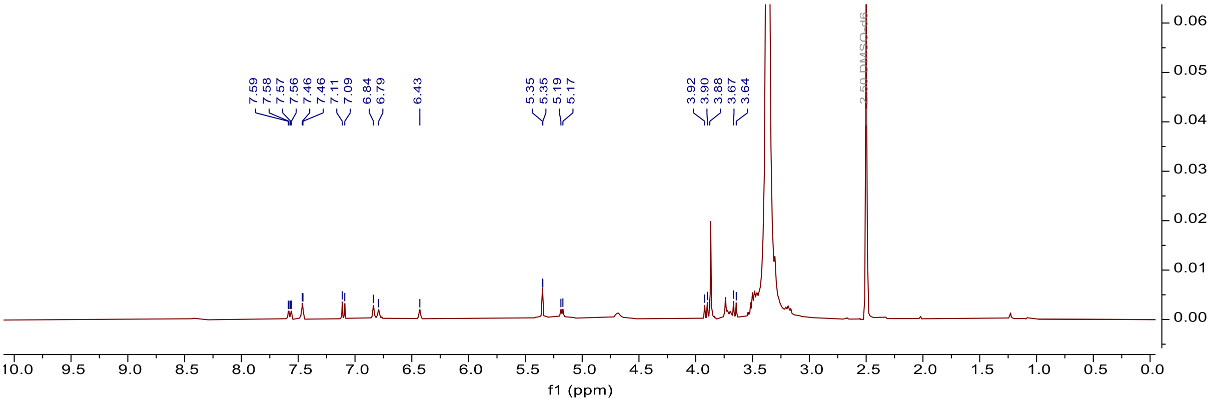

Figure S17. ^{1}H NMR spectrum of compound **5**.

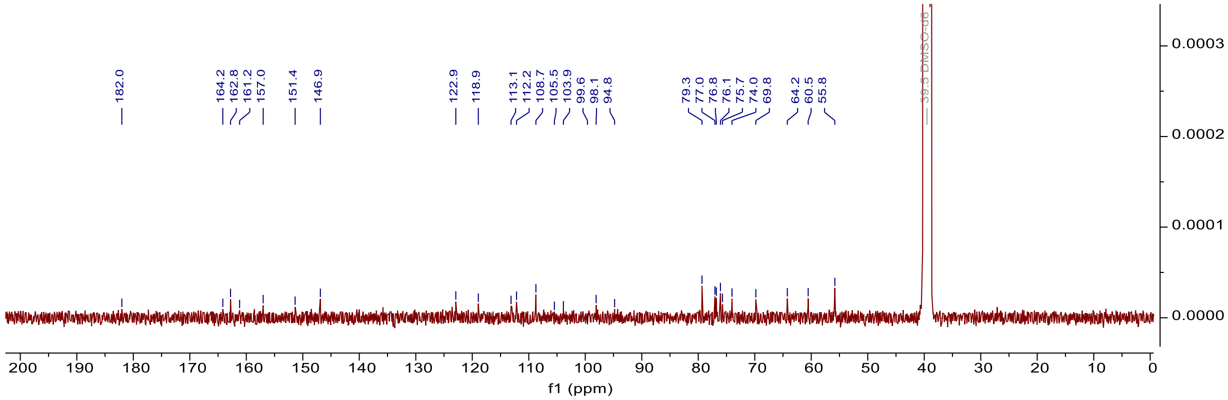

Figure S18. ^{13}C NMR spectrum of compound **5**.

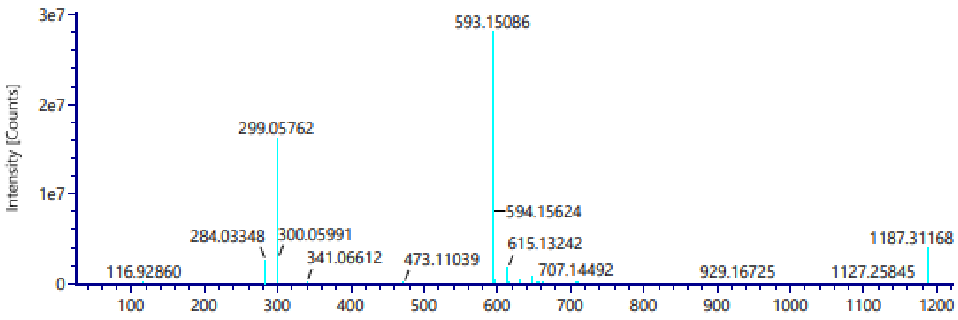

Figure S19. HR-ESI-MS/MS spectrum of compound **5**.

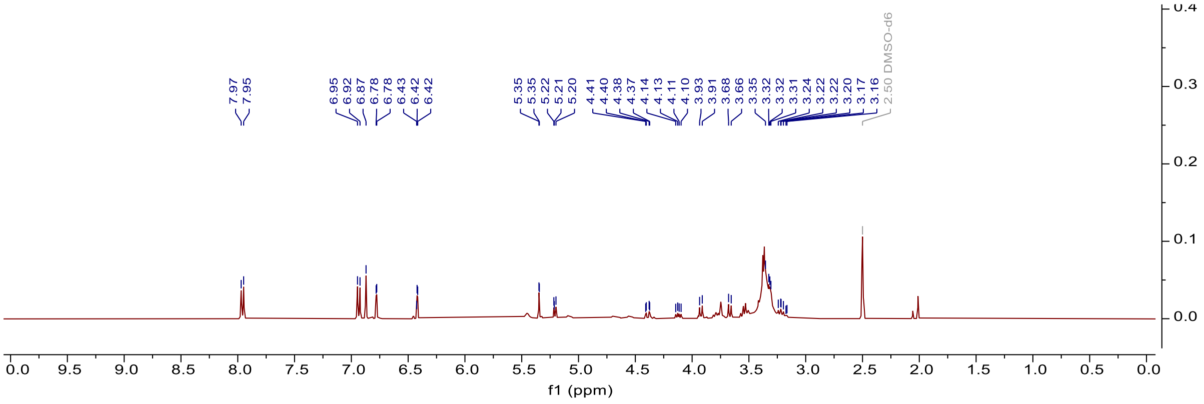

Figure S20. ^{1}H NMR spectrum of compound **6**.

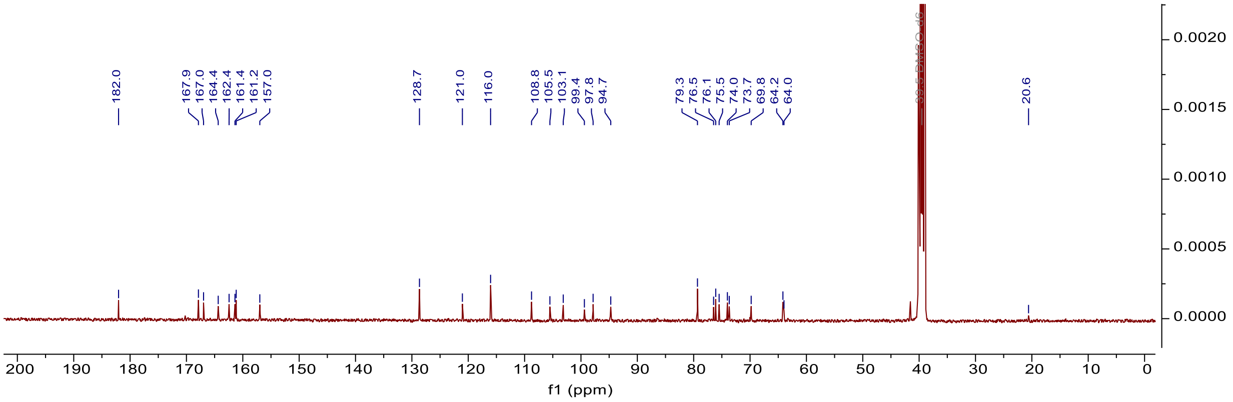

Figure S21. ^{13}C NMR spectrum of compound **6**.

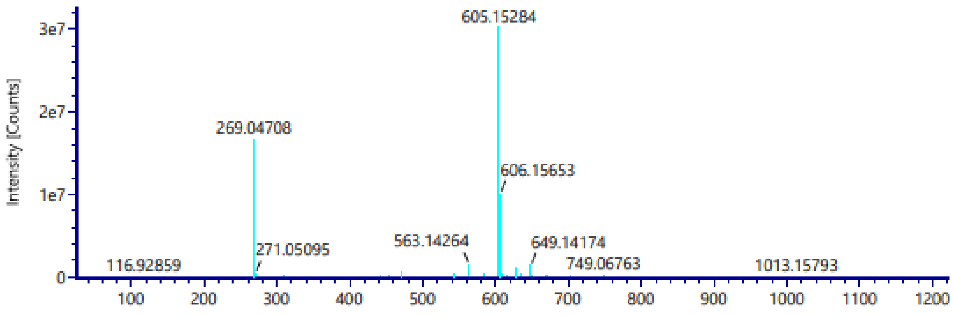

Figure S22. HR-ESI-MS/MS spectrum of compound **6**.

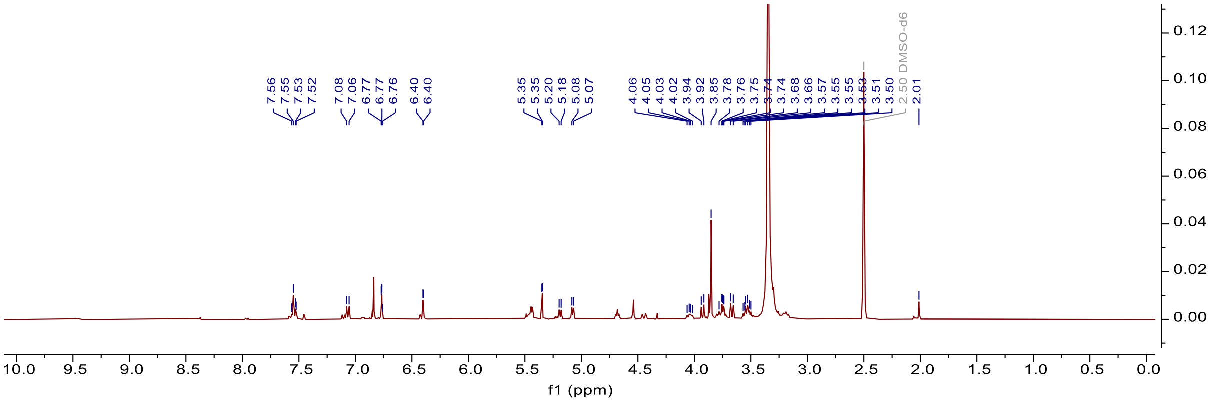

Figure S23. ^{13}C NMR spectrum of compound **7**.

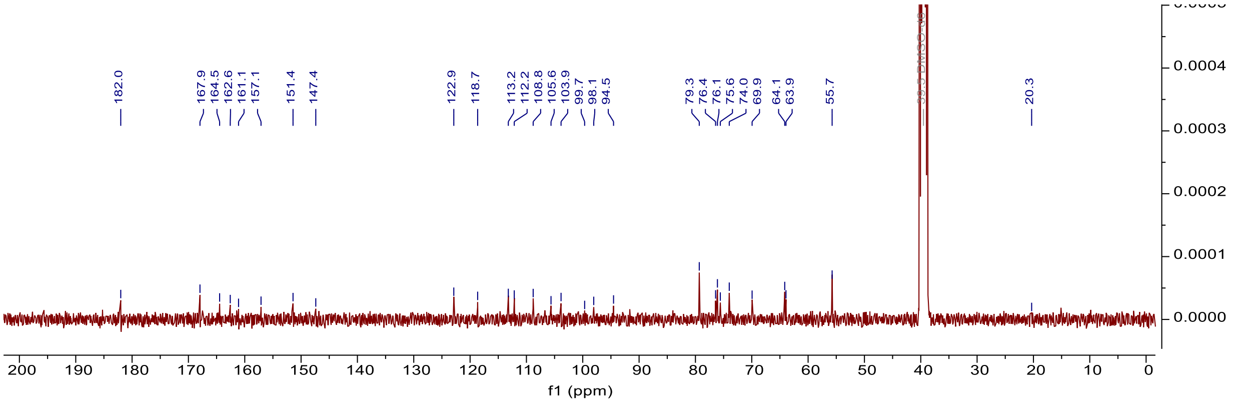

Figure S24. ^{13}C NMR spectrum of compound **7**.

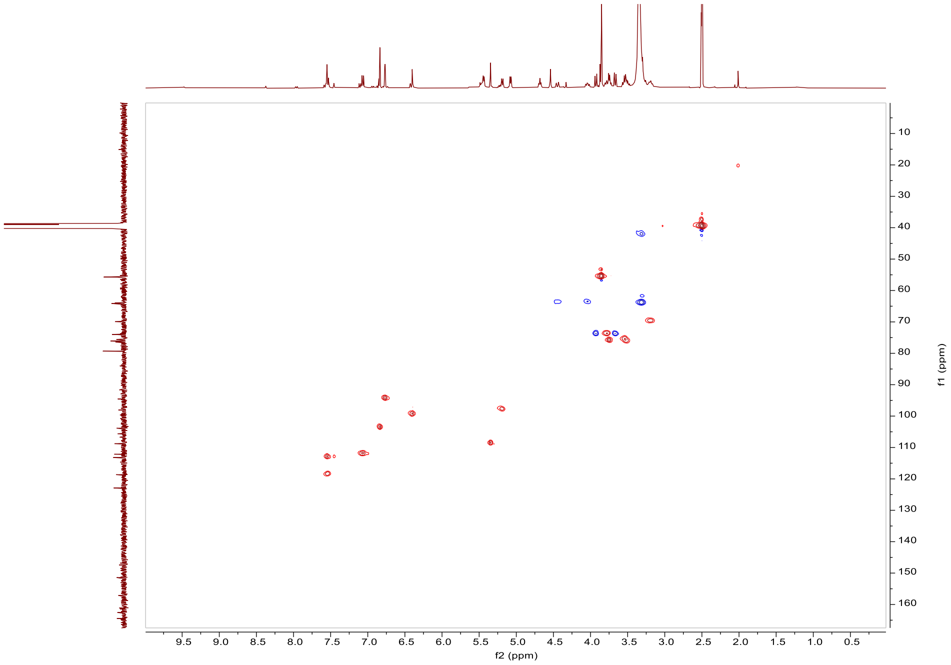

Figure S25. ^{1}H-^{13}C HSQC spectrum of compound **7**.

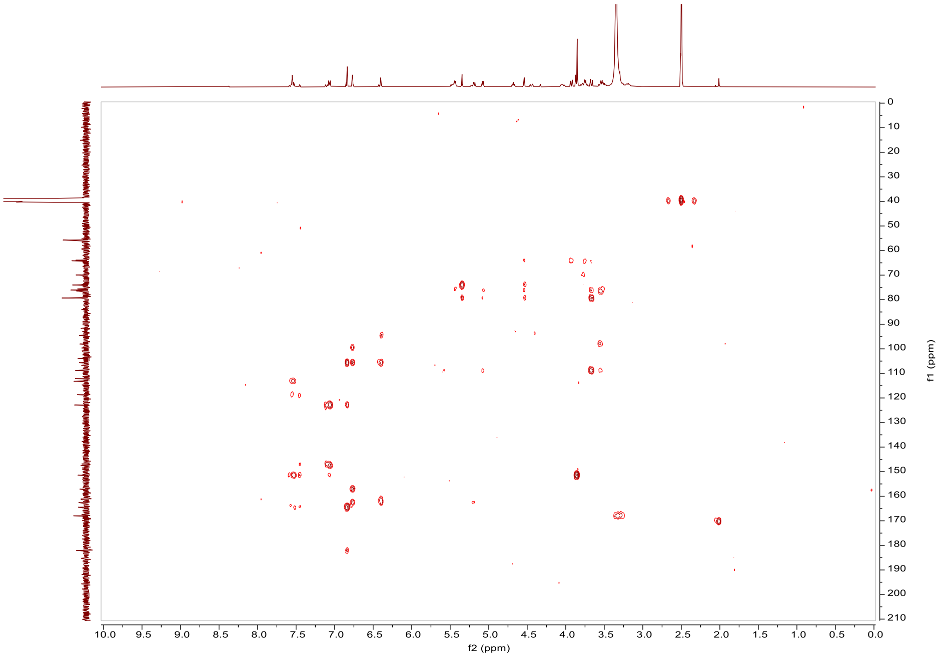

Figure S26. ^{1}H-^{13}C HSQC spectrum of compound **7**.

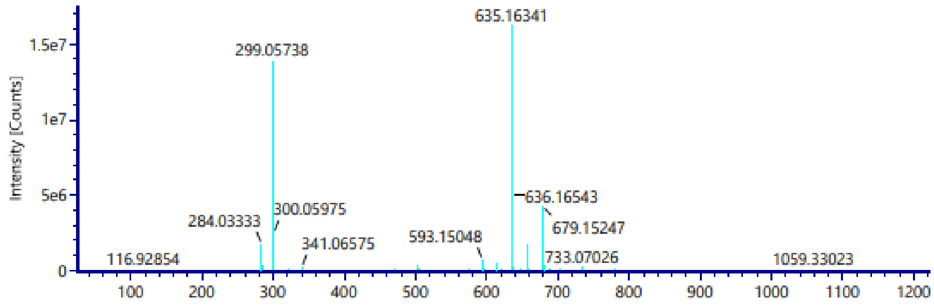

Figure S27. HR-ESI-MS/MS spectrum of compound **7**.

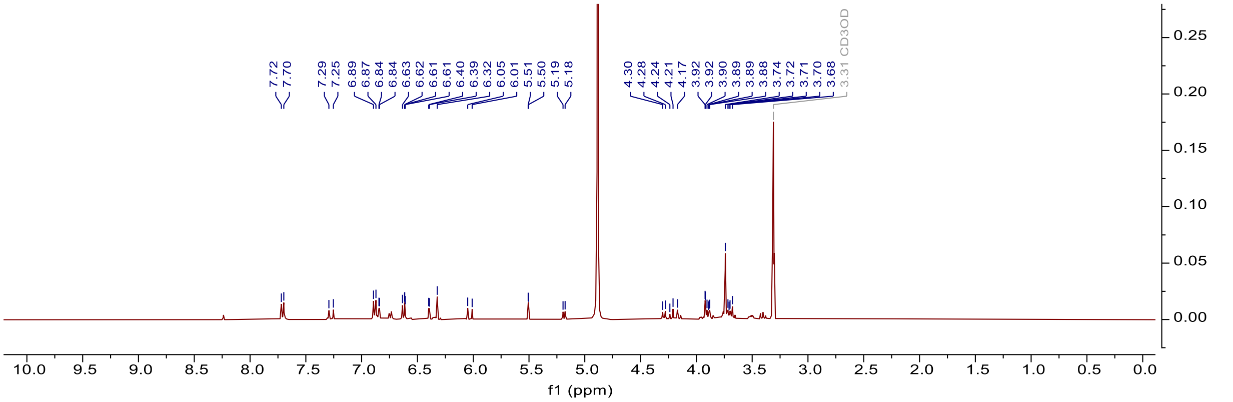

Figure S28. ^{1}H NMR spectrum of compound **8**.

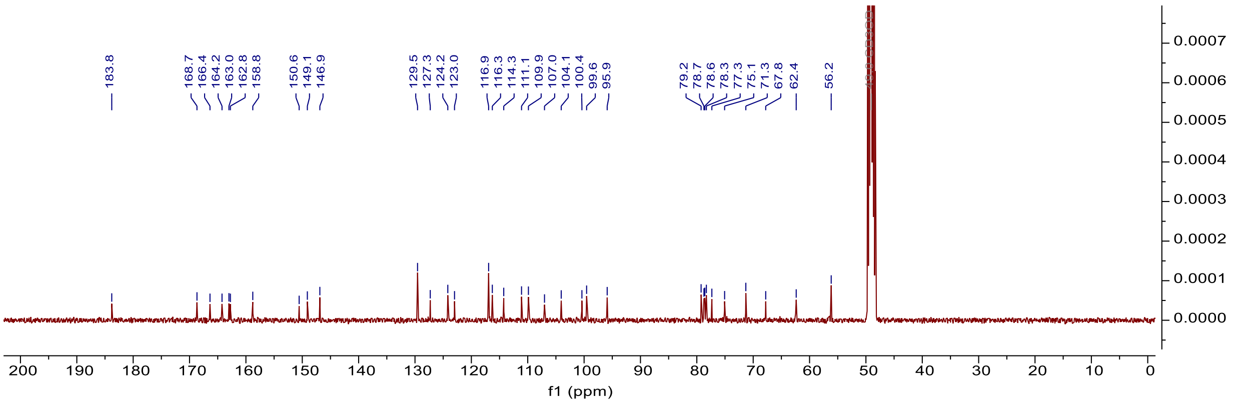

Figure S29. ^{13}C NMR spectrum of compound **8**.

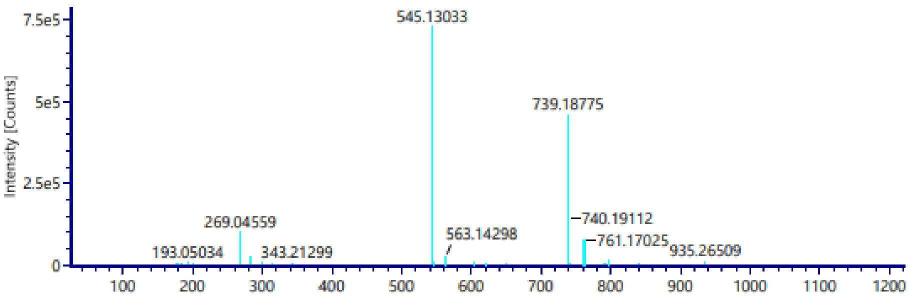

Figure S30. HR-ESI-MS/MS spectrum of compound **8**.

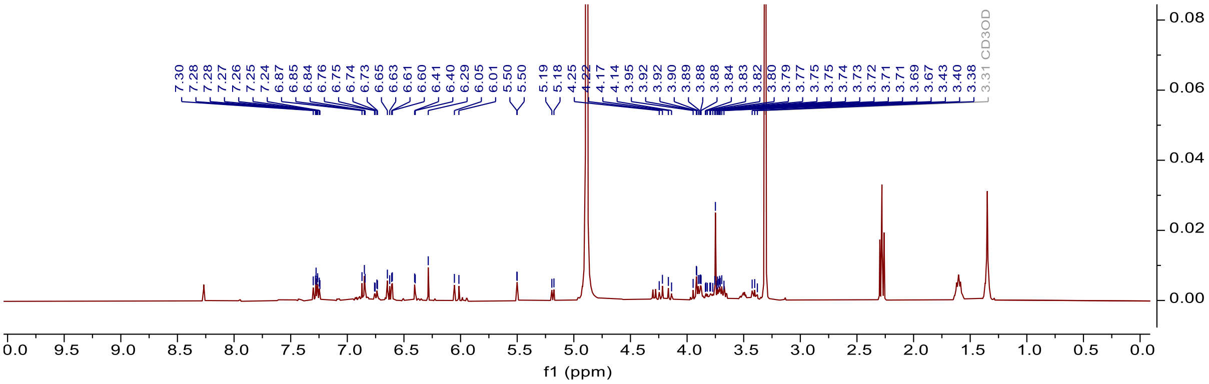

Figure S31. ^{1}H NMR spectrum of compound **9**.

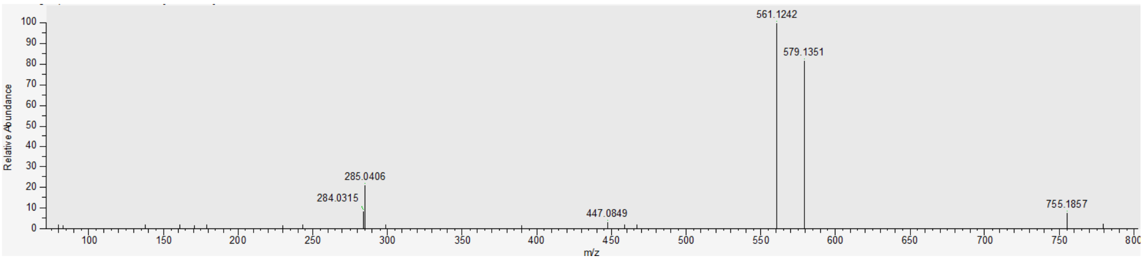

Figure S32. HR-ESI-MS/MS spectrum of compound **9**.

2. In silico studies

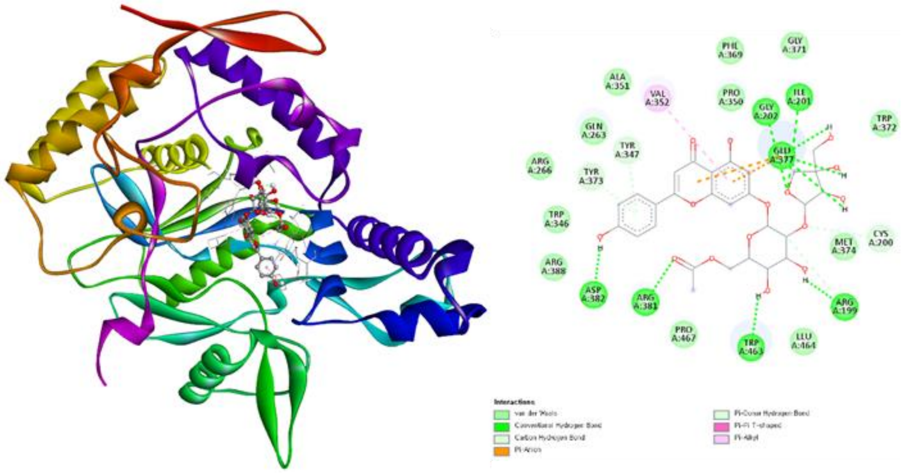

Figure S33. Interactions between binding sites of the iNOS receptor with respect to ligand (compound **6**).

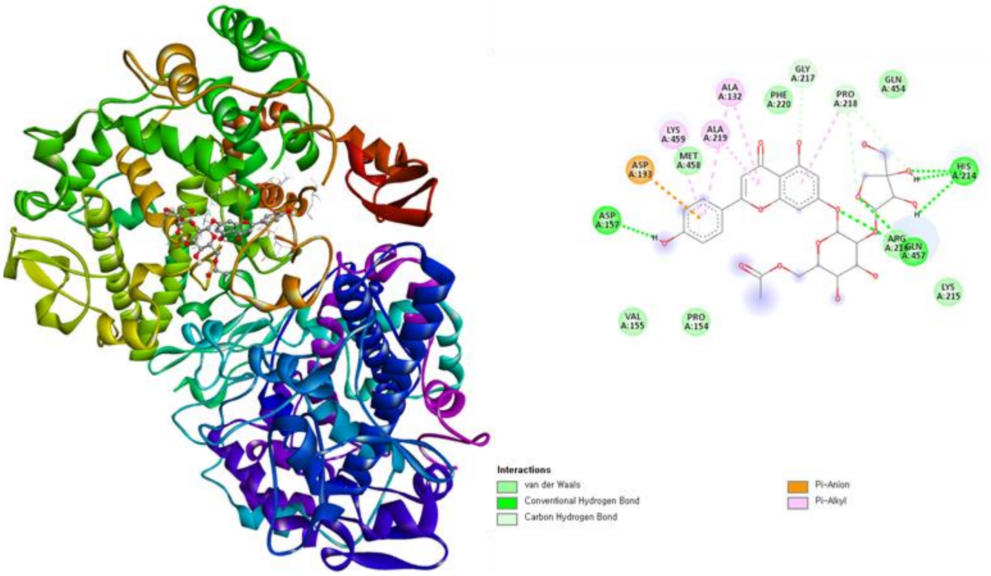

Figure S34. Interactions between binding sites of the COX-2 receptor with respect to ligand (compound **6**).

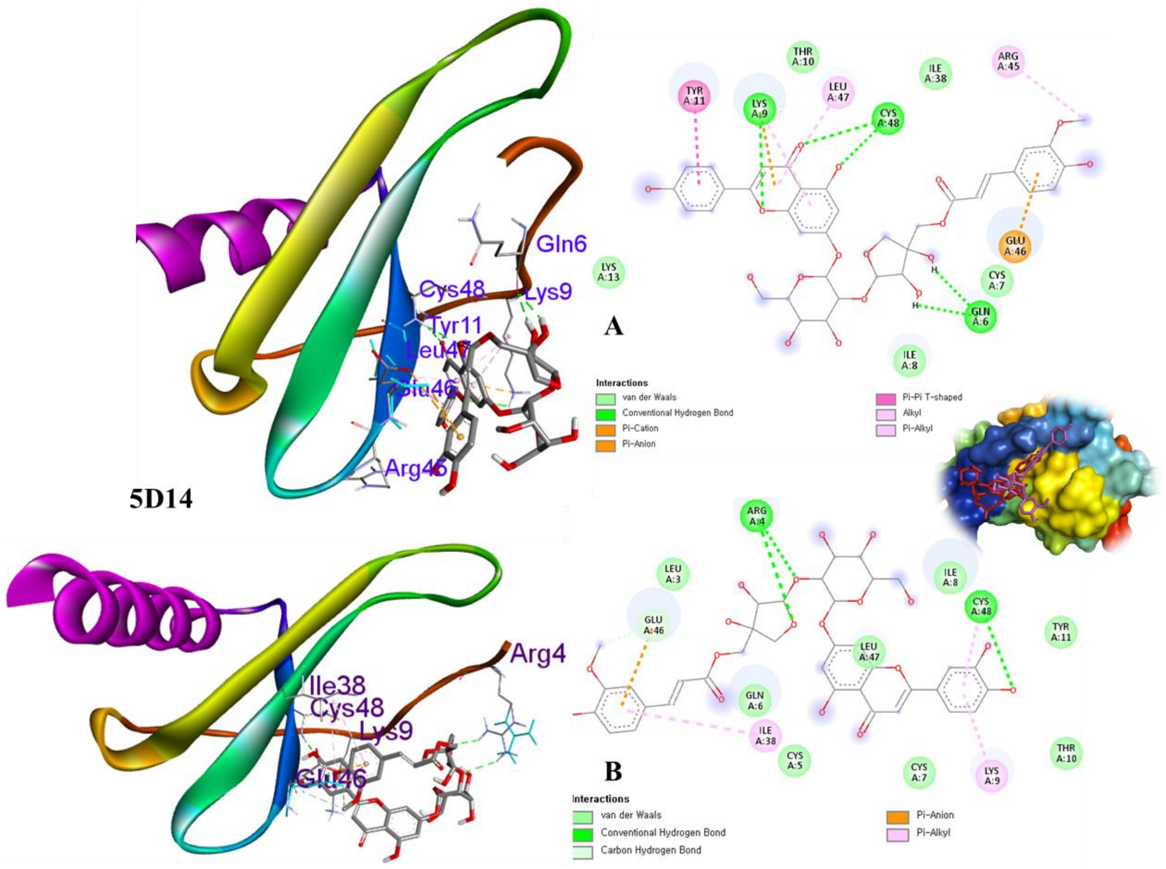

Figure S35. Interactions between binding sites of the IL-8 receptor with respect to ligands [compounds **8** (**A**) and **9** (**B**)].
